# Biomarkers of post-match recovery in semi-professional and professional football (soccer)

**DOI:** 10.3389/fphys.2023.1167449

**Published:** 2023-04-11

**Authors:** Íñigo M. Pérez-Castillo, Ricardo Rueda, Hakim Bouzamondo, José López-Chicharro, Niko Mihic

**Affiliations:** ^1^ Abbott Nutrition, R&D, Granada, Spain; ^2^ Abbott Nutrition, R&D, Chicago, IL, United States; ^3^ Real Madrid, Medical Services, Madrid, Spain

**Keywords:** biomarker, recovery, fatigue, football, soccer, monitoring, metabolomics

## Abstract

High-level football (soccer) players face intense physical demands that result in acute and residual fatigue, impairing their physical performance in subsequent matches. Further, top-class players are frequently exposed to match-congested periods where sufficient recovery times are not achievable. To evaluate training and recovery strategies, the monitoring of players’ recovery profiles is crucial. Along with performance and neuro-mechanical impairments, match-induced fatigue causes metabolic disturbances denoted by changes in chemical analytes that can be quantified in different body fluids such as blood, saliva, and urine, thus acting as biomarkers. The monitoring of these molecules might supplement performance, neuromuscular and cognitive measurements to guide coaches and trainers during the recovery period. The present narrative review aims to comprehensively review the scientific literature on biomarkers of post-match recovery in semi-professional and professional football players as well as provide an outlook on the role that metabolomic studies might play in this field of research. Overall, no single gold-standard biomarker of match-induced fatigue exists, and a range of metabolites are available to assess different aspects of post-match recovery. The use of biomarker panels might be suitable to simultaneously monitoring these broad physiological processes, yet further research on fluctuations of different analytes throughout post-match recovery is warranted. Although important efforts have been made to address the high interindividual heterogeneity of available markers, limitations inherent to these markers might compromise the information they provide to guide recovery protocols. Further research on metabolomics might benefit from evaluating the long-term recovery period from a high-level football match to shed light upon new biomarkers of post-match recovery.

## 1 Introduction

Video monitoring and radio frequency-based time-motion analyses have permitted the characterization of training- and competition-related activity patterns and exercise demands in competitive sports such as football (soccer) (Mohr et al., 2003; Bastida Castillo et al., 2018). Overall, an elite football player covers a distance of 10–13 km during a match (Bangsbo et al., 2006; Collins et al., 2021) at an average intensity close to the anaerobic threshold (≈85% maximum heart rate) (Mohr et al., 2003; Krustrup et al., 2005; Stølen et al., 2005; Collins et al., 2021). Further, high-intensity running activities, such as bursts and sprints, comprise around 10% of match-play time (Gabbett and Mulvey, 2008; O’Donoghue, 2017), which vary depending on playing positions. For example, midfielders cover notably greater distance than central defenders in high-intensity running (Bradley et al., 2009). Other high energy-demanding activities include turnings, tackles, jumping, and various unorthodox movements (i.e., backwards movements, heading, and blocking) which contribute to a relative work rate ≈70–75% maximal oxygen consumption (VO_2_ max) during match-play (Mohr et al., 2005; Bangsbo, 2019). The physical demands derived from football match-play (external load) place a significant physiological strain on the players (internal load), which varies based on their individual characteristics. Compared with in-season training, the loads experienced in match-play are significantly higher (Collins et al., 2021), and players can require up to 72–96 h of recovery time to achieve optimal physical performance in subsequent matches (Ispirlidis et al., 2008; Silva et al., 2018). However, semi-professional and professional players are sometimes exposed to periods of match congestion involving up to eight matches per month, which makes recovery times insufficient (Carling et al., 2015; Noor et al., 2021; Pillay et al., 2022). Adding to the effort of football match-play, players undergo in-season training cycles to prepare for competition (Malone et al., 2015). Adequate planning of training sessions is crucial to optimize training adaptions and avoid non-functional overreaching (Thorpe et al., 2016). To ensure adequate recovery from competition, the monitoring of players’ recovery status is crucial (Nédélec et al., 2012).

Fatigue is a multifactorial concept that involves both central and peripheral mechanisms (Enoka and Duchateau, 2008; Wan et al., 2017; Theofilidis et al., 2018). Muscle fatigue (hereinafter referred to as fatigue) is usually defined as “an exercise-induced reduction in the ability of muscle to produce force or power whether or not the task can be sustained” (Enoka and Duchateau, 2008). High-level players can experience transient fatigue during and after a football match (Mohr et al., 2005). This fatigue usually persists and is characterized by muscle function indicators such as decreased high-intensity running capacity after the most intense 5-min period (Mohr et al., 2003; Bradley et al., 2010; Dalen et al., 2021), lower distance covered in the last compared with the first 15-min period (Mohr et al., 2003; Bradley et al., 2010; Dalen et al., 2021), and poorer physical performance tests scores after the match (Krustrup et al., 2010). Match-induced fatigue might also decrease technical skills during match-play (i.e., shooting and passing) (Russell et al., 2011; Ferraz et al., 2012), yet recent reports in female elite football players suggest that the impact might not be significant (Povoas et al., 2022). Not only decreased physical performance but also increased injury rate has been linked to fatigue (Greig and Siegler, 2009). Accordingly, increased overall risk of injury during match-play has been reported during prolonged congested fixture periods (Dellal et al., 2015; Page et al., 2023). In the same vein, shorter recovery time (≤4 days) has been associated with higher muscle injury rates, particularly hamstring and quadriceps injuries, compared with longer periods (≥6 days) (Bengtsson et al., 2013). Nonetheless, associations of match-induced fatigue and the incidence of various classic football-related muscle injuries warrant further research (Bourne et al., 2019; Huygaerts et al., 2020; Sioud et al., 2023).

**FIGURE 1:**
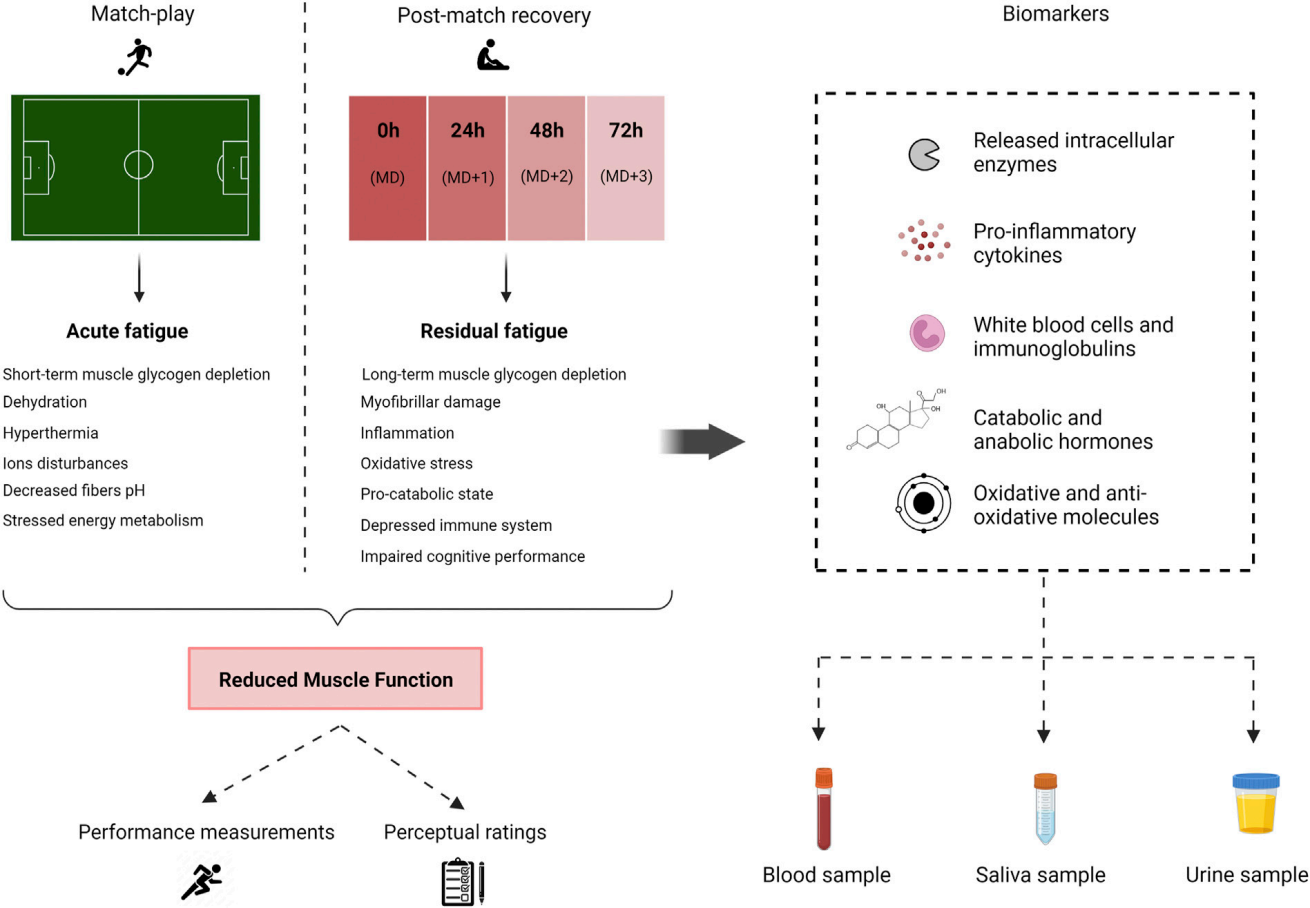
Schematic representation of mechanisms of football match-induced fatigue and potential recovery markers. MD, match-day.

Exercise-induced muscle fatigue is a complex phenomenon that involves multiple underlying mechanisms (Enoka and Duchateau, 2008). Briefly, while in-game fatigue has been proposed to be related to short-term glycogen depletion, dehydration, temperature changes, disturbances in muscle ions, stressed energy metabolism, and decreased fibers pH (Mohr et al., 2005; Bangsbo et al., 2006; Cooke, 2007; Ament and Verkerke, 2009), the recovery period from a football match is defined by processes involving long-term glycogen depletion, exercise-induced muscle damage (EIMD), proinflammatory status, oxidative stress, altered immunity, and disturbances in cognitive performance (Nédélec et al., 2012). Recovery and long-term fatigue involve a non-distinguishable sequence of physiological and psychological factors, which makes the direct observation of the recovery status difficult (Kellmann et al., 2018). Consequently, a wide variety of markers have been proposed to reflect the recovery process, and no single gold-standard marker is available (Finsterer, 2012). Regarding indicators of post-match fatigue, the most frequently used encompass physical performance and subjective measurements as well as the assessment of biochemical analytes (Nédélec et al., 2012). Physical performance measurements evaluate functional aspects, such as sprint performance, jump performance, and muscle function (e.g., knee extensors or lower limb muscles), through the use of batteries of tests (Nédélec et al., 2012; Silva et al., 2018). Concerning subjective markers, these involve the assessment of perceptual ratings [i.e., delayed onset muscle soreness (DOMS)] through visual analogue scales. These tests are routinely used to directly characterize the magnitude of the loss of muscle functionality during postgame and evaluate the stress imposed by football match-play. Nonetheless, these measurements are not exempt from limitations based on the protocol used, including poor reliability, lack of sensitive, and dependence on match contextual factors (Nédélec et al., 2012; Marqués-Jiménez et al., 2017; Carling et al., 2018). To supplement these measurements, biochemical indicators provide objective data on changes in blood, salivary, and urinary analytes that can be particularly useful to guide recovery protocols (Djaoui et al., 2017). A scheme representing all these aspects is presented in Figure 1.

Studying circulating levels of biochemical markers can shed light on the underlying mechanisms involved in post-match recovery. However, as no single biomarker can fully capture the player’s physiological response to football match-play, researchers have explored a range of analytes. Furthermore, there is a need for identifying new biomarkers that overcome limitations of those currently available (Nowakowska et al., 2019). Novel evidence on these molecules has emerged since the latest reviews on the topic (Nédélec et al., 2012; Silva et al., 2018), and the role that metabolomics might play in this field as well as recent efforts aimed to address the high inter-individual variability of already available markers have not been previously reviewed. Therefore, the present narrative review aims to synthetize and discuss the available evidence on analytes that might act as metabolic signatures of post-match recovery in semi-professional, and professional football players, while also providing insights into differences between male and female high-level football players when available. Moreover, strategies used to individualize cutoff values and the applicability of metabolomics to the monitoring of post-match recovery will be disclosed.

## 2 Methods

To provide a comprehensive overview of the current state of the art and discuss recent advancements and future perspectives on the use of biomarkers of football post-match recovery, a non-systematic approach was adopted. An exhaustive search of the scientific databases and search engines ScienceDirect, Web of Science, and Google Scholar was conducted by one reviewer. PubMed (Medline) and EMBASE were also screened using the search tool ProQuest Dialog^®^. Peer-reviewed English-written studies published from inception to December 2022 were screened to identify eligible articles. Relevant search terms (i.e., football, recovery, post-match, EIMD) in combination with Boolean operators (“AND,” “OR,” “NOT”) were used, and references from retrieved articles and previous reviews were screened to further identify other records. Inclusion criteria consisted of studies 1) enrolling male or female semi-professional or professional football players; 2) using official or friendly football matches as exercise protocol, or validated match simulation protocols; 3) reporting data on circulating biochemical markers to evaluate players’ post-match physiological response and/or recovery. Studies conducted in amateur players, and enrolling players with mean age < 16 years were excluded, yet some of these studies were cited and specified when necessary. To help visualize the information, articles reporting fluctuations of these biomarkers after single matches or several matches separated by at last 3 days of recovery time are presented in Supplementary Tables S1–S5.

## 3 Biomarkers of post-match recovery in football

### 3.1 Biomarkers of exercise-induced muscle damage

Football training sessions and competitive matches expose the players to EIMD produced mainly as a consequence of the high eccentric muscle contraction demands that characterize common football-related movements (i.e., changes in direction and speed; backward and sideways running, etc.) (Andersson et al., 2008; Ascensao et al., 2008; Nedelec et al., 2013). Furthermore, increased match scheduling demands predispose elite football players to a higher risk of EIMD compared with amateur football players (Ispirlidis et al., 2008). EIMD is manifested by both immediate and long-term processes. Mechanical loading of the exercised muscle leads to sarcomere overstretching and excitation-contraction (E-C) coupling failure, thus contributing to the primary damage phase. The resulting calcium leakage and the subsequent inflammatory response that occurs during the following days after exercise contribute to the secondary damage phase, which is linked to DOMS (Morgan and Allen, 1999; Howatson and van Someren, 2008). Importantly, EIMD occurrence has been linked to impaired muscle gluconeogenesis (Costill et al., 1990) and is known to associate with reduced muscle power generation, as denoted by common markers of muscle function such as reduced intermittent sprint performance (Twist and Eston, 2005), squat jump performance (Byrne and Eston, 2002a), and knee extensor isometric strength (Byrne and Eston, 2002b). The membrane disturbances resulting from EIMD release muscle proteins to the blood stream which can be detected and quantified thus acting as potential biomarkers. As post-match fatigue is proposed to partially derive from EIMD, the analysis of EIMD biomarkers might provide useful insights into the recovery process (Marqués-Jiménez et al., 2017).

Serum creatine kinase (CK) is the most commonly measured analyte in the assessment of EIMD as greatest increases in serum CK activity are produced in response to intensive eccentric compared to concentric contractions (Newham et al., 1986; Halson, 2014). CK is an enzyme found in cytosol and mitochondria of tissues subject to high energy demands. It plays an important role in the regeneration of cellular adenosine triphosphate (ATP) through the reversible phosphorylation of creatine to phosphocreatine and the stabilization of ATP at the expense of phosphocreatine (Wallimann et al., 2011; Baird et al., 2012). It is known that serum CK levels increase following exercise performance, although peak serum CK values are observed at different time points depending on the type of exercise (Totsuka et al., 2002).

Regarding football, a vast number of studies conducted in male and female semi-professional and professional football players have reported values of CK as a means to assess EIMD in training and competition (Andersson et al., 2008; Gravina et al., 2011; Rampinini et al., 2011; Thorpe and Sunderland, 2012; Gatterer et al., 2013; Gunnarsson et al., 2013; Nedelec et al., 2013; Silva et al., 2013; Souglis et al., 2015a; Pimenta et al., 2015; Russell et al., 2015; Mohr et al., 2016; Bouzid et al., 2018; Henrique et al., 2018; Marques-Jimenez et al., 2018; Souglis et al., 2018; Sparkes et al., 2018; Botelho et al., 2020; Pooley et al., 2020; Chou et al., 2021; Csala et al., 2021; Daab et al., 2021; Garcia-Romero-Perez et al., 2021; Hacker et al., 2021; Khaitin et al., 2021; Kostrzewa-Nowak et al., 2021; Trecroci et al., 2021; Selmi et al., 2022). Of note, as susceptibility to EIMD is higher among players unaccustomed to eccentric exercise, returning to high-intensity training and competition from off-season and periods of recovery from injury are considered critical time points for monitoring (Nédélec et al., 2012). Accordingly, CK assessment has been explored in the monitoring of EIMD during preseason, a period characterized by intensive training programs aimed to adapt the players to competition demands (Pimenta et al., 2015; Coppalle et al., 2019; Botelho et al., 2020; Radziminski et al., 2020; Selmi et al., 2022).

**FIGURE 2:**
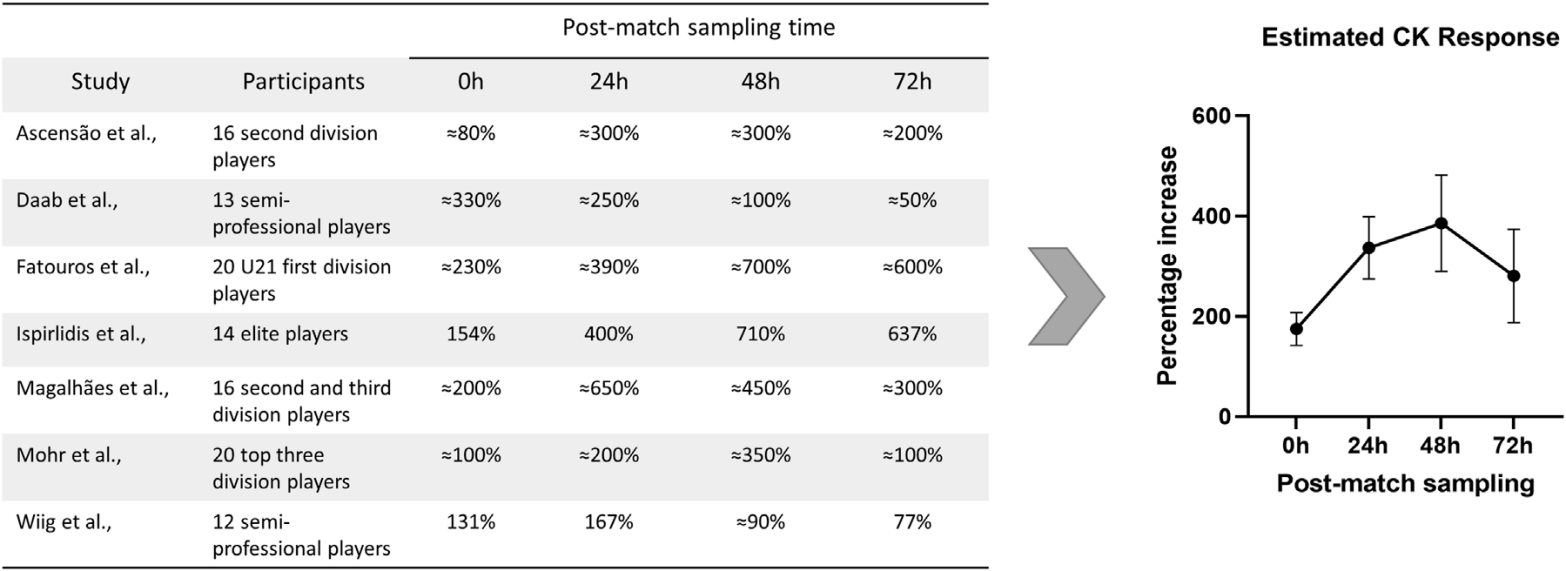
Data from studies reporting percentage increases in circulating CK levels of male football players during post-match recovery. Data presented as percentage increase during recovery compared with pre-match values. The estimation of the average CK response was calculated by pooling CK percentage increases as mean ±SEM at the different time-points. CK, creatine kinase; SEM, standard error of the mean. Citations: (Ascensao et al., 2008; Daab et al., 2021; Fatouros et al., 2010; Ispirlidis et al., 2008; Magalhães et al., 2010; Mohr et al., 2016; Wiig et al., 2022).

Besides high-intensity training periods, important increases in circulating CK levels immediately after a match or a simulated football match test are typically regarded as a sign of EIMD produced during match-play (Andersson et al., 2008). Several studies have explored the impact of football match-play on blood CK levels of male players at different time points. Accordingly, CK levels have been reported to peak at 24 h postgame (Rampinini et al., 2011; Silva et al., 2013; Bouzid et al., 2019; Wiig et al., 2022) and gradually decrease throughout the recovery period, with elevated CK levels persisting up to 48–72 h after the match or match simulation (Ascensao et al., 2008; Rampinini et al., 2011; Silva et al., 2013; Daab et al., 2021; Wiig et al., 2022). Alternatively, other time-course analyses have observed CK levels peaking at 48 h postgame (Ispirlidis et al., 2008; Fatouros et al., 2010; Mohr et al., 2016) or have reported different deviations to these dynamics (Gunnarsson et al., 2013; Fransson et al., 2018; Bouzid et al., 2019). Observed peak values are highly heterogeneous with some authors reporting 2-4-fold increases (Rampinini et al., 2011; Gunnarsson et al., 2013; Bouzid et al., 2018; Fransson et al., 2018; Daab et al., 2021; Wiig et al., 2022) while others have documented more drastic increases in circulating CK levels compared to pre-match values (Ispirlidis et al., 2008; Fatouros et al., 2010; Mohr et al., 2016). Factors such as previous exposure to intensive exercise might lead to lower elevations in CK levels resulting from increased enzyme clearance (Hyatt and Clarkson, 1998). In Figure 2 are presented data estimated or extracted from studies evaluating circulating CK levels of male football players after a football match or match simulation test.

Regarding periods of match-congestion, higher CK levels are typically observed along with increasing number of matches played. On this subject, Garcia-Romero-Perez et al. (2021) showed that pre-match CK levels are significantly higher among players exposed to > 60 min of match-play during congested compared to non-congested periods, thus representing an important aspect to consider when deciding on availability of players across matches (Garcia-Romero-Perez et al., 2021). Chou et al. (2021) reported in female players that three and six Loughborough Intermittent Shuttle Tests (LIST) played in consecutive days induced a higher increase in CK blood levels compared to a single LIST, which was suggestive of accumulation of muscle damage across tests (Chou et al., 2021). However, the recovery rate remained unchanged, which was proposed to denote attenuated magnitude of EIMD over multiple LISTs as a consequence of the repeated bout effect (Chou et al., 2021). Notably, although increases in postgame CK levels have been reported as a maker of EIMD in elite female football players (Andersson et al., 2008; Gravina et al., 2011; Chou et al., 2021), male players might display higher CK increases compared to female players after football games probably due to differences in the number of eccentric actions performed, and a potential protective role of estrogens in muscle cellular membranes (Mougios, 2007; Souglis et al., 2018). Besides sex, a number of different factors might act as potential confounders when evaluating associations between postgame CK levels and EIMD in football, namely, playing positions, with midfielders having higher postgame levels compared to defenders (Malone et al., 2018; Souglis et al., 2018; Nowakowska et al., 2019; Freire et al., 2021), ethnicity (Mahmutyazicioglu et al., 2018), genetic factors (Pimenta et al., 2012), and training status, which might contribute to the elevated interindividual heterogeneity of CK levels across semi-professional and professional football players (Baird et al., 2012).

Several efforts have been made to address the high inter-individual variability in CK data. Bayesian statistics utilizes prior knowledge in the form of prior distributions and combines it with observational data to determine the posterior distribution, thereby predicting future outcomes (van de Schoot et al., 2021). Using a Bayesian strategy, Hecksteden et al. (2017) collected CK levels at recovered and non-recovered states in athletes of different sport disciplines with the purpose of creating individualized cutoff values (Hecksteden et al., 2017). Error rates for CK values were significantly lower when using the individualized classification, which was proposed to improve accuracy of monitoring post-exercise recovery (Hecksteden et al., 2017). This approach was shown successful for the monitoring of CK values in elite badminton players (Barth et al., 2019), and in a recent study in professional football players (Skorski et al., 2022). In particular, individualized CK values were shown to display significantly lower error rates when differentiating recovered (one day off) and non-recovered state (two strenuous training sessions within two days) compared with group-based analysis in 163 male German fourth division players (Skorski et al., 2022). While this approach shows promise for monitoring individual CK values in athletes, it may not be intuitive for professionals to implement, and the requirement for prior individualized values can present a challenge. Lastly, based on previous research focused on associations between external load metrics and CK values at team level (Hader et al., 2019; Wiig et al., 2019), models aimed to predict individualized CK values have been recently reported (Schuth et al., 2022). Identifying which load parameters best correlate this marker is important (Schuth et al., 2022).

Another biochemical analyte whose serum levels increase after physical exercise as a sign of muscle damage is lactate dehydrogenase (LDH). LDH constitutes a group of oxidoreductase isoenzymes (LDH-1, LDH-2, LDH-3, LDH-4, and LDH-5) present in almost all body tissues, which plays an important role in energy metabolism as it catalyzes the reversible conversion of pyruvate to lactate with the concomitant conversion of NADH to NAD^+^ (Khan et al., 2020). Parallel to CK levels, total circulating LDH activity levels increase notably after prolonged physical exercise, with eccentric contractions inducing higher increases compared to concentric contractions (Brancaccio et al., 2008). Compared with CK, LDH levels have been suggested to peak earlier in response to intensive exercise (Reichel et al., 2020; Tokinoya et al., 2020). In high-level male football players, total LDH blood levels have been reported to increase right after official matches or match simulation tests (Ispirlidis et al., 2008; Souglis et al., 2015a; Trajković et al., 2018; Daab et al., 2021), with circulating levels peaking during the following 13–24 h (Souglis et al., 2015a; Daab et al., 2021), and remaining sustained during the second and/or the third day after the match (Ispirlidis et al., 2008; Marques-Jimenez et al., 2018; Daab et al., 2021; Moya-Amaya et al., 2021). Nonetheless, deviations to these dynamics have been also documented (i.e., peak levels occurring at 0 or 48 h postgame) (Ispirlidis et al., 2008; Parsaie et al., 2019). In addition, several studies reporting elevated LDH blood values at 18–24h and 48 h after football matches have been conducted in female players (Gravina et al., 2011; Souglis et al., 2018; Bonilla et al., 2020), who might display lower match-induced LDH increases compared to male players (Souglis et al., 2018). Like CK, circulating levels of LDH might also fluctuate due to different interindividual factors (Brancaccio et al., 2008). The lack of specificity of LDH to the myocyte challenges its use as an indirect marker of EIMD, leading to a preference for CK as a more reliable marker (Sorichter et al., 1999).

Besides CK and LDH, some studies have reported circulating levels of aspartate transaminase (AST) and alanine transaminase (ALT) [formerly known as oxaloacetic transaminase (GOT) and glutamic pyruvic transaminase (GPT)] to assess EIMD in male and female elite football players (Bassini-Cameron et al., 2007; Gravina et al., 2011; Becatti et al., 2017; Marques-Jimenez et al., 2018; Trajković et al., 2018; Nowakowska et al., 2019; Radziminski et al., 2020; Clemente et al., 2021; Mitrotasios et al., 2021; AdibSaber et al., 2022). Although typically linked to liver pathologies, the release of AST and ALT from skeletal muscle cells can occur as a response to intensive physical exercise (Lippi et al., 2011; Chamera et al., 2014). Furthermore, it has been proposed that the activity of these enzymes might correlate to the intensity and duration of the physical effort (Chamera et al., 2014). Accordingly, peak serum levels of AST, and ALT have been shown to occur up to 96 h after acute eccentric exercise protocols (Kanda et al., 2014). Regarding football studies, significant increases in AST levels (≈35%) were reported in U21 Croatian players immediately after an official match (Trajković et al., 2018). Elite male football players in a placebo intervention trial showed comparable increases in ALT and AST immediately after the match and 24 h later, as well as elevated levels of CK and LDH, indicating match-induced muscle damage (AdibSaber et al., 2022). However, inconsistent results have been reported in different studies. For instance, Gomes et al. (2018) reported inconsistent changes in ALT and AST activity immediately and 48 h after two scheduled football matches separated by three days in professional football players (Gomes et al., 2018). In the same vein, Gravina et al. (2011) did not observe changes in ALT and AST levels neither immediately nor 18 h after a football match in high-level female football players. On the other hand, clear increases in LDH and CK were reported (Gravina et al., 2011). Overall, time-course analyses of these enzymes during the post-match recovery period are scarce. Lastly, other muscle proteins such as myoglobin leak to the systemic circulation from skeletal muscle after damage-inducing exercises. However, although myoglobin levels are typically reported to increase immediately after football matches and simulated football matches as a sign of EIMD (Ascensao et al., 2008; Thorpe and Sunderland, 2012; Gunnarsson et al., 2013; Fransson et al., 2018; Trajković et al., 2018; Wiig et al., 2022), levels tend to normalize at 24 h post-match (Ascensao et al., 2008; Gunnarsson et al., 2013; Fransson et al., 2018), thus being of limited utility for the monitoring of EIMD during post-match recovery.

Based on these studies, supporting the assessment of functional and perceptual fatigue measurements with individualized ranges of CK, and/or combinations of different EIMD biomarkers such as LDH might overcome limitations inherent to individual markers and provide useful insights into the monitoring of post-match recovery.

### 3.2 Biomarkers of inflammation

A key consequence of the structural disarrangements of the skeletal muscle membranes produced during the early stages of EIMD consists of the accumulation of calcium ions. This subsequently triggers an inflammatory response proposed to be involved in different aspects of muscle repairing and remodeling processes (Peake et al., 2017). The time-course of the inflammatory response will depend on the type, intensity, and duration of the exercise performed and thus sport-specific patterns may exist. For example, football postgame inflammatory response has been reported to be larger than that of other team sports such as handball, volleyball, and basketball (Clarkson and Hubal, 2002; Souglis et al., 2015a).

As part of the acute-phase of the inflammatory process, cytokines are produced by leucocytes and the muscle itself (the latter being termed “myokines”) (Pedersen et al., 2007; Paulsen et al., 2012), some of which are typically reported as inflammatory biomarkers. Among cytokines, circulating levels of interleukine-6 (IL-6), a cytokine generally recognized as pro-inflammatory, have been consistently reported to increase after intensive exercise due to skeletal muscle secretion without requiring the presence of EIMD (Chan et al., 2004; Paulsen et al., 2012). Nonetheless, secondary increases derived from EIMD can be produced (Hennigar et al., 2017). Moreover, IL-6 is known to play important roles in energy metabolism (Chan et al., 2004) and has been suggested to correlate with fatigue sensation after intensive exercise (Proschinger and Freese, 2019).

As IL-6 levels might reflect the need for glycogen replenishment and might further increase following EIMD, authors have resourced to the analysis of this cytokine to characterize the inflammatory response produced during the post-match recovery process (Parsaie et al., 2019; Trecroci et al., 2021). Among them, Ispirlidis et al. (2008) analyzed serum IL-6 levels in male elite football players participating in a football match and compared them with control players. The authors observed that post-match IL-6 concentrations measured 2 h after the match were strongly increased in comparison with pre-match and control levels, and normalized 24 h afterwards (Ispirlidis et al., 2008). These results are aligned with those obtained by Romagnoli et al. (2016) in professional football players, observing notably increased IL-6 levels at 30 min postgame compared to base levels, which also returned to resting values 24 h afterwards (Romagnoli et al., 2016). Even stronger increases in IL-6 circulating levels were reported by Souglis et al. (2015a), right after an official match, which remaining slightly elevated at 13 h postgame (Souglis et al., 2015a). Similarly, Mohr et al. (2016) measured IL-6 levels in professional male football players participating in three football games within 1 week, collecting the samples before and at 0, 24, 48, and 72 h after each game. Accordingly, IL-6 levels were shown to increase right after each match and return to base values during the following 24 h, albeit differences were not statistically significant. These dynamics remained unchanged across different matches (Mohr et al., 2016). More recently, Koziol et al. (2020) reported almost 6-fold increased IL-6 values immediately after a competitive match in male U19 Polish players, which returned to resting values after 24 h (Koziol et al., 2020). Increases in IL-6 values have also been consistently reported in elite female players with levels returning to base values at 24 h after the match (Andersson et al., 2010a; Souglis et al., 2015b; Souglis et al., 2018). While some studies have reported no differences in postgame IL-6 dynamics between male and female football players (Souglis et al., 2015b), others have observed lower peak values in women compared to male players for all positions (Souglis et al., 2018).

IL-6 is produced in larger amounts compared to any other cytokine in response to high intensity exercise. The initial release of IL-6 usually precedes a less pronounced increase in the levels of different pro- and anti-inflammatory cytokines, with IL-1β and tumor necrosis factor α (TNF-α) being the most widely studied (Pedersen et al., 1998; Pedersen, 2000). In particular, concentrations of IL-1β have been observed to increase during post-match (Ispirlidis et al., 2008; Mohr et al., 2016; Jatene et al., 2019) and throughout the competitive season (Nobari et al., 2021) in elite football players, yet studies are scarce and inconsistent changes in circulating values have been reported (Andersson et al., 2010a; Hacker et al., 2021). In the same vein, TNF-α is another pro-inflammatory cytokine, which is speculated to play a role in muscle regeneration (Yang and Hu, 2018), and whose serum levels have been documented to peak immediately after a football match, with postgame concentrations being ≈100%–200% higher when compared to pre-game values (Souglis et al., 2015a; Koziol et al., 2020). Nonetheless, Andersson et al. (2010a) analyzed the cytokines response of elite female football players participating in two friendly football games separated by 72 h of active or passive recovery observing that increases in TNF-α occurred only during the first match postgame. On the other hand, IL-6 levels were shown to increase immediately after both matches (Andersson et al., 2010a). Aligned with other markers, postgame TNF-α peaks have been reported to be significantly lower in female compared to male football players which might partially explain differences in inflammatory response between both sexes (Souglis et al., 2015a).

C-reactive protein (CRP) is a protein synthetized by the hepatocytes, whose circulating levels are markedly impacted by acute inflammatory stimuli (Black et al., 2004). In this regard, chronic physical exercise reduces CRP levels while short-term strenuous exercise leads to transient increases in CRP activity produced by an inflammatory acute phase response mediated by IL-6 and IL-1β (Zhang et al., 1995; Kasapis and Thompson, 2005). The physiological perturbations produced during football matches and match simulations seem to trigger an acute-phase inflammatory response denoted by increased CRP levels. Notably, while increases in the concentrations of the already commented cytokines (IL-6, IL-1β, and TNF-α) tend to occur during the immediate postgame, increments in circulating CRP have been consistently reported to peak around 24 h after the match (1.5- to 3-fold increases) (Ispirlidis et al., 2008; Silva et al., 2013; Souglis et al., 2015b; Mohr et al., 2016; Romagnoli et al., 2016; Fransson et al., 2018; Daab et al., 2021; Duarte et al., 2022), being base values restored within 72 h of recovery time in male elite football players. Regarding female players, similar peak levels have been reported 13–24 h postgame (Gravina et al., 2011; Souglis et al., 2015b; Souglis et al., 2018; Goulart et al., 2021). In addition, CRP circulating levels have been proposed to also be sensitive to training loads and match-congestion periods throughout the competitive season (Anđelković et al., 2015; Coppalle et al., 2019; Jatene et al., 2019; Duarte et al., 2022; Selmi et al., 2022). However, discrepancies in different studies have been reported probably due to methodological heterogeneity (Saidi et al., 2021). Interestingly, some authors have suggested that increases in CRP due to EIMD might require body contact to occur (Wiewelhove et al., 2015).

In summary, the postgame response of cytokines such as IL-6 seems to reflect adaptative changes and the muscle’s attempt to restore homeostasis following intensive exercise while increments in CRP levels may characterize the secondary inflammatory process as a consequence of muscle damage induced by match-play. While changes in IL-6 levels seem to be rather acute, monitoring post-match CRP levels might assist couches and health professionals in the monitoring of match-induced inflammatory process during post-match recovery, and can supplement the information provided by other markers.

### 3.3 Biomarkers of immune response

Both male and female athletes competing in highly aerobic sports typically display substantially lower white blood cells (WBC) counts compared with standard clinical reference values at rest (Horn et al., 2010; Diaz Martinez et al., 2022). However, prolonged and high intensity endurance exercise produce important shifts in WBC counts denoted by transitory increases in circulating granulocyte (primarily neutrophil) and monocyte counts, and decreases in lymphocytes populations (Nieman, 2000), which can persist hours and days into the recovery period. While acute exercise induces immediate increases in neutrophil and lymphocyte counts (Kurokawa et al., 1995; Shek et al., 1995), the recovery periods is denoted by a decrease in circulating lymphocyte levels, with NK cell levels being reduced even several days after exercise cessation (Shek et al., 1995; Simpson et al., 2007).

These changes in WBC dynamics in response to exercise have been proposed to be consequence of different factors including cytokines release (Yamada et al., 2002) and secretion of stress-related hormones such as catecholamines (Kappel et al., 1991), growth hormone (Kappel et al., 1993), and cortisol (TØNnesen et al., 1987). As hypothesized by some authors, if lymphocytopenia remains during a further exercise bout an “open window” of immunodepression might occur thus increasing the risk of infection in athletes (Gleeson, 2002). Furthermore, in the presence of EIMD, leukocytes mobilize to the injured muscle playing a critical role in muscle repair and regeneration processes (Chazaud, 2016). Following the initial neutrophiles response, phagocytic macrophages are recruited to the muscle, leading to the removal of cellular debris and the release of cytokines, proteases, and oxidative compounds required to coordinate muscle repair (Tidball and Villalta, 2010; Ziemkiewicz et al., 2021). In fact, blocking the activity of neutrophils and the recruitment of macrophages to the muscle has been shown to impair the regeneration of the injured tissue (Teixeira et al., 2003; Martinez et al., 2010). Therefore, monitoring fluctuations in immune cell counts and distributions in response to a football match has been suggested to provide useful insights into the player’s immune status during the recovery period.

Total WBC counts have been consistently reported to increase ≈20–100% within the first hour after a football match or match simulation in male and female elite players (Malm et al., 2004; Ascensao et al., 2008; Ispirlidis et al., 2008; Andersson et al., 2010a; Fatouros et al., 2010; Magalhães et al., 2010; Gravina et al., 2011; Mohr et al., 2016; Romagnoli et al., 2016; Poulios et al., 2018; Trecroci et al., 2021). Changes in leukocyte circulating levels are mainly dependent on neutrophilia, with neutrophil counts being ≈50%–200% increased immediately after the match (Malm et al., 2004; Ascensao et al., 2008; Andersson et al., 2010a; Gravina et al., 2011; Romagnoli et al., 2016; Trecroci et al., 2021), yet elevations in monocyte counts have also been documented (Romagnoli et al., 2016; Trecroci et al., 2021). On the other hand, while some authors have reported unchanged lymphocyte counts during the immediate postgame (Romagnoli et al., 2016; Trecroci et al., 2021), others have observed substantial decreases in lymphocyte counts right after a football match (Ascensao et al., 2008; Magalhães et al., 2010; Gravina et al., 2011).

Match-induced perturbations in total WBC counts are usually restored within 24–48 h of recovery time (Ascensao et al., 2008; Fatouros et al., 2010; Magalhães et al., 2010; Gravina et al., 2011; Mohr et al., 2016; Romagnoli et al., 2016). Nonetheless, periods of match-congestion might place a strain on players’ immune system denoted by decreased populations of leukocytes days long into recovery. Malm et al. (2004) evaluated the impact that playing two football matches within two consecutive days has on different leukocyte populations in elite players. The observed that while total leukocyte and neutrophile counts were notably increased immediately after the second match, lymphocyte and monocyte levels were substantially lower compared with resting values (Malm et al., 2004). Remarkably, total leukocyte counts were shown to decrease substantially below resting values during the following 24 h. Moreover, natural killer lymphocyte counts remained substantially reduced up to 48 h after the second game, which in combination with alterations in signaling and adhesion molecules was suggested to be indicative of decreased immunity (Malm et al., 2004). This is aligned with ensuing studies reporting impaired immune function in professional football players during periods of match-congestion (Heisterberg et al., 2013). Longer recovery periods seem to be sufficient to restore the immune function. Accordingly, Mohr et al. (2016) did not observed total WBC counts below resting values at any time point in professional players participating in a normal microcycle consisting of three matches separated by three and four recovery days (Mohr et al., 2016). Similarly, playing two football matches separated by 72 h of recovery time was shown not to decrease leukocyte counts below resting values in different studies (Andersson et al., 2010a; Poulios et al., 2018).

Besides white cells, immunoglobulins have received great attention as markers of immune status in sports. Among them, immunoglobulin A (IgA) is the most abundant immunoglobulin in external secretions thus constituting the first line of defense against invading pathogens (Woof and Kerr, 2006). IgA assessment has attractive advantages over WBC counts as it can be measured in saliva, a non-invasive sample with better applicability in the context of sports practice, and lower salivary IgA (sIgA) levels have been linked to higher incidence of upper respiratory tract infections (URTI) in athletes (Neville et al., 2008; Mortatti et al., 2012). Recent reviews have reported that sIgA levels tend to decrease in response to training loads and congested schedules in different sport disciplines and football (Rico-González et al., 2021a; Rico-González et al., 2021b). However, some studies evaluating the impact of competitive exercise on sIgA levels have reported inconsistent findings (Campbell and Turner, 2018). In fact, absolute IgA levels have been observed to decrease right after a football match (Penailillo et al., 2015), increase as a response to two bouts of football-specific exercises (Sari-Sarraf et al., 2007), and remain unchanged during immediate postgame (Moreira et al., 2009). Similarly, while different studies have reported significant decrements in sIgA levels (Morgans et al., 2014; Mortatti et al., 2020; Mortatti et al., 2022), others have observed no alterations in sIgA in response to match-congested periods (Page et al., 2019) or successive matches (Maya et al., 2016). Recently, Morgans et al. (2022) reported a trend towards increased sIgA levels from pre-to post-match in male elite players (Morgans et al., 2022); however, a decrease was noted from the day before the match to 60 min before kick-off, which was concluded to suggest stress caused by preparation for official match-play (Morgans et al., 2022).

As reviewed by Campbell and Turner (2018), decrements in lymphocyte counts seem not to necessarily represent immunosuppression after physical exercise as lymphocytopenia might be indicative of peripheral mobilization of these immune cells, which might be beneficial for immune surveillance (Campbell and Turner, 2018). In addition, associations between sIgA and URTI are unclear and differences in reporting of IgA levels [i.e., correcting for saliva flow rate and differences in sampling procedure (Lindsay and Costello, 2017)] and inter-individual variation (i.e., oral health status) might preclude drawing solid conclusions (Campbell and Turner, 2018). Although the role that changes in WBC counts and immunoglobulin levels plays in post-match recovery seems unclear, the impact of match-congested periods on players immune status is a topic that deserves further research.

### 3.4 Biomarkers of endocrine response

Physical exercise constitutes a stressing situation that threatens to alter body homeostasis (Mastorakos et al., 2005). Consequently, central and peripheral mechanisms are activated in order to provide a functional response to the stress stimulus (Mastorakos et al., 2005). The hypothalamic-pituitary-adrenal axis (HPA) is a major regulator of the neuroendocrine response to exercise-induced physical and psychological stress and is responsible for the release of cortisol, the main hormone related to the stress response (Dedovic et al., 2009). Cortisol mediates wide physiological processes denoted by immunosuppression (Shinkai et al., 1996), enhanced gluconeogenesis (Khani and Tayek, 2001), and increased catabolism [i.e., increased protein breakdown (Brillon et al., 1995), and lipolysis (Divertie et al., 1991)]. Therefore, elevated cortisol levels have been proposed to decrease sport performance due to the impairment of muscle strength derived from an increased catabolic state (Ispirlidis et al., 2008). On the other hand, testosterone is a major anabolic hormone produced in the gonads, which plays an important role in muscle protein synthesis, particularly in response to resistance exercise (Hooper et al., 2017). Testosterone is also known to favor glycogen replenishment and reduce protein breakdown in muscle (Herbst and Bhasin, 2004; Kelly and Jones, 2013), thus counterbalancing the catabolic actions of cortisol.

Early increases in testosterone levels following exercise are proposed to be caused by decreased plasma clearance (Cadoux-Hudson et al., 1985) while subsequent increases have been typically attributed to the action of cortisol, although a causative association remains unclear (Brownlee et al., 2005). Levels of both cortisol and testosterone are responsive to exercise practice and might vary depending on training level (Jiménez et al., 2020), modality (Vanhelder et al., 1985), intensity (Leicht et al., 2018), and duration (Brownlee et al., 2005) of the exercise performed. Thus, several authors have resourced to the analysis of cortisol, testosterone, and the testosterone/cortisol ratio (T:C) to characterize the sport-specific anabolic/catabolic balance of athletes during recovery from training and competition (Adlercreutz et al., 1986; Elloumi et al., 2003; Anderson et al., 2016). Furthermore, these hormones can be measured in saliva samples, which largely mirror circulating levels and can be assessed in a non-invasive manner (Gatti and De Palo, 2011).

Athletes typically display peak serum and salivary cortisol levels minutes after high-intensity exercise (Paccotti et al., 2005; Minetto et al., 2007). Extrapolated to football research, studies conducted in the 90s already reported significant increases in serum cortisol levels of semi-professional male football players during the half-time and immediately after a football match, with concentrations returning to base values within the following 90 min (Lupo et al., 1985; Carli et al., 1986). Ensuing studies have reported similar findings with serum and salivary cortisol levels being elevated during the immediate postgame (Malm et al., 2004; Ispirlidis et al., 2008; Souglis et al., 2015a; Jatene et al., 2019; Pinto et al., 2021; Morgans et al., 2022), although serum cortisol levels have also been documented to remain increased during the following 24 and 48 h in some cases (Silva et al., 2013; Mohr et al., 2016; Morgans et al., 2022), or even at 72 h postgame (Marqués-Jiménez et al., 2022). On the contrary, non-significant changes or even decrements in postgame cortisol levels have also been observed (Penailillo et al., 2015; Romagnoli et al., 2016). Regarding testosterone, studies assessing total and free testosterone levels right after football matches have usually reported significant decrements compared to pre-match values (Lupo et al., 1985; Malm et al., 2004; Penailillo et al., 2015; Romagnoli et al., 2016; Jatene et al., 2019), albeit inconsistencies are also present in the literature (Ispirlidis et al., 2008; Thorpe and Sunderland, 2012; Mohr et al., 2016). Nonetheless, the use of the T:C ratio, which has been proposed to denote physiological strain in the context of overtraining (Urhausen et al., 1995; Urhausen and Kindermann, 2002), appears to be unreliable when assessing alterations in the anabolic/catabolic balance during recovery from a football match (Silva et al., 2013; Penailillo et al., 2015; Romagnoli et al., 2016).

Changes in cortisol levels might reflect the psychophysiological stress induced by match-congested periods, which in turn might impair physical performance in subsequent matches (Clarke et al., 2009). Mohr et al. (2016) observed a significant increase in cortisol levels in one out of three matches (24 h after the second match) separated by 72 h of recovery time with no consequent impact on testosterone levels (Mohr et al., 2016). On the contrary, non-significant changes in salivary cortisol levels were observed across four matches played within four consecutive days in U17 male football players (Pinto et al., 2021). Comparisons between congested and non-congested competitive schedules have revealed unaltered salivary cortisol levels in elite players (Owen et al., 2019; Saidi et al., 2020), while decreases in testosterone and T:C ratio were documented in one study (Saidi et al., 2020). Similar conclusions have been reached in younger football players (under 16 years old) subjected to a congested match schedule (Moreira et al., 2016).

Research on hormonal changes in response to elite female football matches is scarce and differences owing to menstrual cycle phases and the lower physiological levels of testosterone in women are to be expected. On this subject, immediate increases in serum free testosterone levels in response to a football match were reported by Gravina et al. (2011) in female players (Gravina et al., 2011). On the contrary, unchanged or decreased salivary free cortisol levels along with decrements in salivary free testosterone levels were reported by Casanova et al. (2016) after all encounters during an elite football competition (Casanova et al., 2016), which seem not to correlate with performance measurements (Casanova et al., 2019). Besides sexual differences, match-induced changes in cortisol and testosterone values might be influenced by wide different aspects including players’ circadian rhythm (Teo et al., 2011; Sparkes et al., 2018), timing of post-match samples collection (important fluctuations might occur within 15–30 min into the postgame) (Lupo et al., 1985), contextual factors [i.e., home advantage (Fothergill et al., 2017), winning vs. losing (Oliveira et al., 2009), anticipatory cortisol response (Oliveira et al., 2009)], and hydration status (Castro-Sepulveda et al., 2018). Moreover, other methodological and technical issues related to the collection of saliva samples such as stimulation of salivation, previous dietary and water intake, and oral health status should also be considered (Hayes et al., 2016).

Overall, current evidence suggests that football matches are capable of inducing immediate endocrine responses in professional and semi-professional male football players as denoted by increased cortisol and decreased testosterone levels. However, most of the studies conducted to date have evaluated acute changes in hormones levels in response to a football match while evidence on long-term effects is inconsistent and seems insufficient to recommend the monitoring of hormones during the post-match recovery period.

### 3.5 Biomarkers of oxidative status

Free radicals are molecules or fragments of molecules possessing an unpaired electron (Cheeseman and Slater, 1993). Among these, reactive oxygen species (ROS) and reactive nitrogen species (RNS) are the most representative primary free radicals and are implicated in the regulation of immune processes and skeletal muscle adaptation mechanisms (Finaud et al., 2006). During skeletal muscle contraction, primary free radicals such as superoxide, nitric oxide, hydroxyl radicals, and hydrogen peroxide, among others, are generated from oxygen metabolism in mitochondria and produced through enzymatic reactions (i.e., NADPH oxidases and phospholipase A2), a process countered by enzymatic and non-enzymatic antioxidative systems resulting in a pro-oxidant/antioxidant redox balance (Powers et al., 2020). Disturbances in this balance skewed towards an increased pro-oxidative state induce the oxidation of cellular components such as lipids, proteins, and nucleic acids, which leads to the formation of secondary oxidation products and promotes mechanisms of inflammation and cellular apoptosis (Finaud et al., 2006). EIMD might further contribute to the exacerbated production of oxidative species (Lee et al., 2002) while oxidative stress might also aggravate the EIMD process (Duarte et al., 1993). Furthermore, while low-level oxidative molecules seem to increase force production, oxidative stress is thought to be implicated in the loss of muscle force associated to exercise-induced fatigue (Reid et al., 1994; Smith and Reid, 2006).

Postulated mechanisms by which free radicals might reduce muscle force production are multiple and englobe impairment of membrane excitability through inhibition of the sodium-potassium-ATPase-pump (McKenna et al., 2006), inhibition of the sarcoplasmic reticulum calcium-ATPase activity (Gutiérrez-Martín et al., 2004), and decreased myofibrillar calcium sensitivity (Andrade et al., 2001; Moopanar and Allen, 2005), among others [underlying mechanisms have been extensively reviewed elsewhere (Powers and Jackson, 2008)]. Indeed, it is well-established nowadays that various types of physical exercise including both prolonged endurance exercise and eccentric exercise can induce oxidative stress evidenced by increased circulating levels of oxidative molecules that might act as biomarkers (Powers et al., 2020). Nonetheless, although changes in levels of oxidative compounds in response to a bout of exercise are rather acute (Michailidis et al., 2007), oxidative stress also contributes to the acute-phase inflammatory response resulting from EIMD, and thus biomarkers of oxidative stress are susceptible to be elevated days after a match in football players (Fatouros et al., 2010). This delayed oxidative stress response has been proposed to mainly derive from neutrophils and phagocytic cells activity, which might be influenced by higher susceptibility of damaged tissue to oxidative processes, and decreased clearance of circulating oxidants (Nikolaidis et al., 2007; Nikolaidis et al., 2008).

**FIGURE 3:**
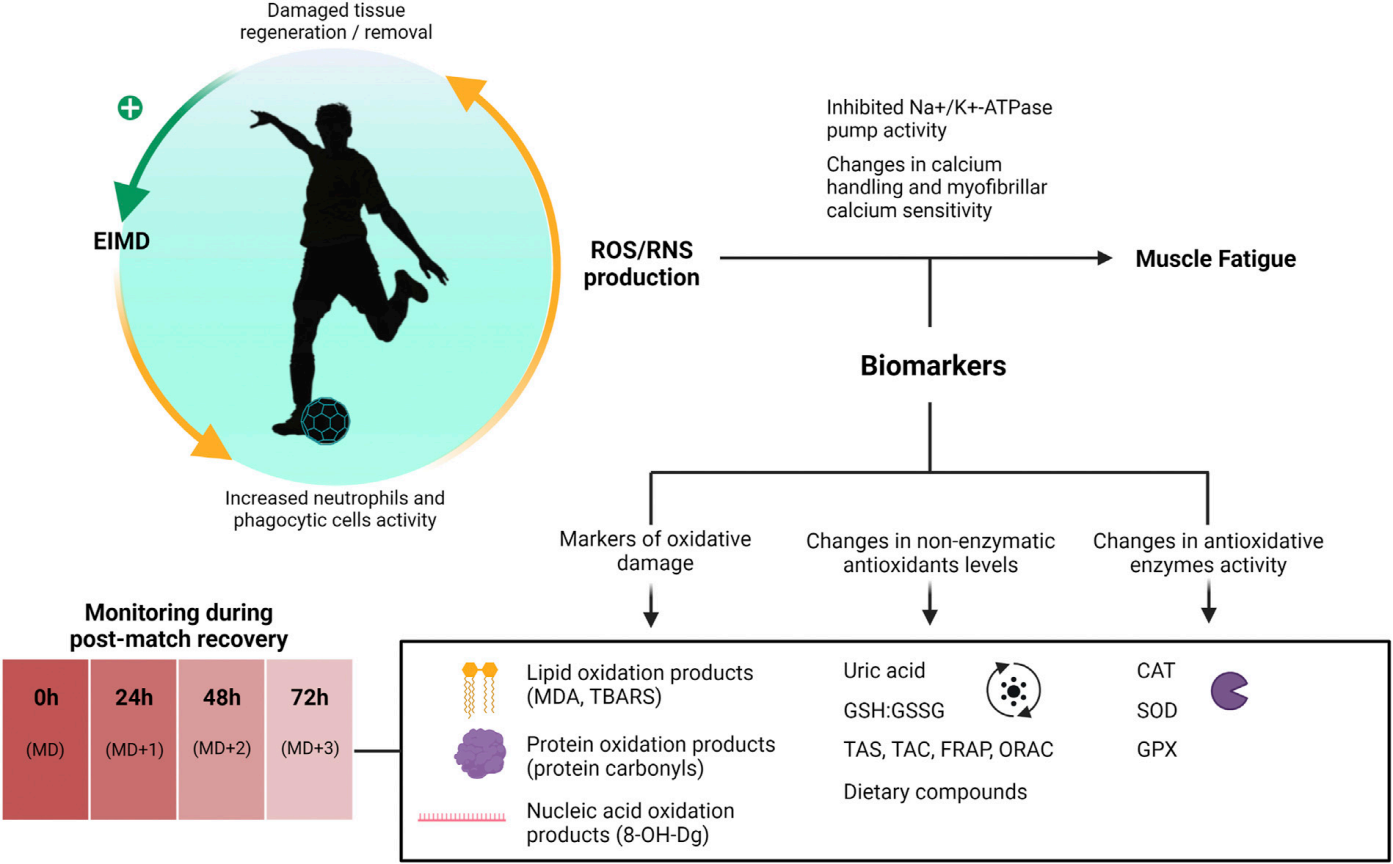
Biomarkers of oxidative status during post-match recovery. 8-OH-Dg, 8-hydroxy-2-deoxyguanosine; CAT, catalase; EIMD, exercise-induced muscle damage; FRAP, ferric reducing antioxidant potential; GHS:GSSG, glutathione to glutathione disulfide ratio; GPX, glutathione peroxidase; MD, Match-day; MDA, malondialdehyde; ORAC, oxygen radical antioxidant capacity; RNS, reactive nitrogen species; ROS, reactive oxygen species; SOD, superoxide dismutase; TAC, total antioxidant capacity; TAS, total antioxidant status; TBARS, thiobarbituric acid reactive substances.

Numerous molecules have been explored as biomarkers of oxidative stress with different degrees of evidence, being typically categorized into primary oxidants, oxidated molecules, antioxidative molecules, and antioxidative enzymes (Frijhoff et al., 2015; Marrocco et al., 2017). In the context of sports research, the direct analysis of free radicals (ROS and RNS) faces complex methodological limitations that preclude its practical application (Kalyanaraman et al., 2012). Alternatively, authors have resourced to the analysis of molecules resulting from the oxidation of cellular components caused by oxidative damage. Protein carbonyls are the most commonly reported biomarker of irreversible oxidation of proteins (Bloomer et al., 2007a; Bloomer et al., 2007b) while malondialdehyde (MDA) and thiobarbituric acid reactive substances (TBARS) (Spirlandeli et al., 2014) are frequently used to estimate lipid oxidation, and 8-hydroxy-2-deoxyguanosine (8-OH-Dg) reflects nucleic acids oxidation processes (Larsen et al., 2020). Similarly, training and competition are proposed to modulate the activity of antioxidative enzymes such as superoxide dismutase (SOD), catalase (CAT) and glutathione peroxidase (GPX), which can be quantified following exercise (Powers et al., 1994; Selamoglu et al., 2000; Aguiló et al., 2005). Lastly, the assessment of other antioxidant molecules such as glutathione (GSH) and the reduced glutathione to glutathione disulfide ratio (GSH:GSSG) (Elokda et al., 2005) as well as the assessment of general non-enzymatic antioxidant molecules such as uric acid (UA) o exogenous antioxidant molecules (i.e., vitamin C and E) might provide useful insights into redox balance after exercise (Gomez-Cabrera et al., 2021). The role that ROS/RNS play in muscle fatigue and potential biomarkers involved are illustrated in Figure 3.

UA is an end-product of purines metabolism that denotes nucleotide turnover and acts as a potent scavenger of reactive species *in vivo* thus contributing substantially to plasma total antioxidant capacity (Grootveld and Halliwell, 1987; Glantzounis et al., 2005), albeit the concept of UA as a mere antioxidant molecule has been challenged (Kurajoh et al., 2021). In football research, UA is frequently used to report increased purines metabolism and compensatory changes in antioxidant capacity in response to match-play. In male elite football players, plasma UA levels have been reported to increase ≈10%–70% immediately after a football match or simulated football match (Ascensao et al., 2008; Magalhães et al., 2010; Bouzid et al., 2019; Trecroci et al., 2021) while other authors have observed no changes during the immediate postgame (Ispirlidis et al., 2008; Fatouros et al., 2010; Silva et al., 2013; Colombini et al., 2014; Souglis et al., 2018). These changes in postgame UA levels have been shown to directly correlate with total antioxidant capacity measurements in some studies (Ascensao et al., 2008; Fatouros et al., 2010). Time-course analyses exploring various types of exercise have observed that changes in circulating UA levels can remain elevated days after exercise performance (Chatzinikolaou et al., 2010; Jamurtas et al., 2018). In fact, several authors have documented elevated UA levels 48–96 h after a football match (Ascensao et al., 2008; Ispirlidis et al., 2008; Fatouros et al., 2010) and 24 h after a LIST (Bouzid et al., 2019), yet inconsistent changes during the recovery period have also been observed (Magalhães et al., 2010; Silva et al., 2013; Souglis et al., 2018). Regarding female players, some authors have documented increased UA levels after a football match (Andersson et al., 2008; Andersson et al., 2010b; Andersson et al., 2010c; Gravina et al., 2011), which returned to base values within a day of recovery time (Andersson et al., 2010b; Andersson et al., 2010c). Differences might not only depend on sex but also on dietary intake, match intensity, and playing positions (Magalhães et al., 2010; Souglis et al., 2018). Lastly, the analyzed sample type is also important as salivary UA measurements have been shown to yield highly inconsistent results in relation to football-related exercises (Gomes et al., 2018; Rodrigues de Araujo et al., 2018).

A major non-enzymatic antioxidant present in all human cells is glutathione (GSH), a tripeptide which is oxidized to glutathione disulfide (GSSG) in the presence of free radicals in a reaction catalyzed by GPX. Glutathione reductase can then catalyze the regeneration of GSH from GSSG using NADPH as an electron donor (Powers and Jackson, 2008). Thus, GSH and GSSG serve as a redox pair sensible to oxidative stress which has also been suggested as a biomarker of overtraining (Margonis et al., 2007). Although GSH levels and the GSH/GSSG ratio are consistently reported to decrease during football post-match recovery (Ascensao et al., 2008; Fatouros et al., 2010; Mohr et al., 2016; Souglis et al., 2018; Koziol et al., 2020) results from studies conducted in male and female players evaluating the time-course of these changes are heterogenous and difficult to compare (Andersson et al., 2008; Andersson et al., 2010b; Andersson et al., 2010c; Fatouros et al., 2010; Mohr et al., 2016; Souglis et al., 2018; Koziol et al., 2020). In general, a decrease in the GSH/GSSG ratio within 24–48 h after a football match might be indicative of oxidative stress associated to match-induced inflammatory processes. In addition, different methods and procedures permit the quantification of the total non-enzymatic antioxidant capacity in body fluids including the assessment of total antioxidant status (TAS), total antioxidant capacity (TAC), ferric reducing antioxidant potential (FRAP), and oxygen radical antioxidant capacity (ORAC), which are quite extended in studies conducted in high-level football players (Ascensao et al., 2008; Andersson et al., 2010b; Fatouros et al., 2010; Magalhães et al., 2010; Costa et al., 2011; Gravina et al., 2011; Silva et al., 2013; Mohr et al., 2016; Gomes et al., 2018). However, these measurements are subject to criticism as methods are diverse, unspecific, and neglect the contribution of intracellular antioxidative enzymes (Bartosz, 2010; Marrocco et al., 2017).

Changes in the total activity of antioxidative enzymes such as CAT, SOD, and GPX have been explored to assess oxidative stress in response to exercise (Urso and Clarkson, 2003). CAT activity is usually reported to increase right after football matches and return to base values within hours in male and female players (Fatouros et al., 2010; Mohr et al., 2016; Souglis et al., 2018). Regarding GPX and SOD values, results are less consistent. Fatouros et al. (2010) reported increases in whole blood GPX activity 24–72 h after a football match with values peaking between 24 and 48 h which was proposed to be dependent on scavenger cell migration to the damaged fiber (Fatouros et al., 2010). Similarly, Mohr et al. (2016) observed higher GPX activity compared with baseline and control players, which remained elevated up to 72 h after three football matches separated by 3 days (Mohr et al., 2016). On the contrary, Silva et al. (2013) documented decreases in GPX activity 24 h after a football match (Silva et al., 2013). Similarly, unchanged SOD and GPX values were observed by Gravina et al. (2011) in female players (Gravina et al., 2011). Besides enzymatic activity, Koziol et al. (2020) observed that the serum protein levels of the isoforms CuZnSOD/SOD1 and GPX1 remained unchanged at any time-point in U19 players participating in a competitive football match (Koziol et al., 2020). However, these isoforms are mainly located in the cytosol and measuring their serum protein levels might be of limited utility. Based on these mixed results, relying only in the monitoring of blood antioxidative enzymes activity seems insufficient to evaluate redox status during post-match recovery.

The blood levels of lipid peroxidation by-products reflect oxidative damaged inflicted to muscle cell membranes probably as a consequence of the production of free radicals by phagocytic cells (Fisher-Wellman and Bloomer, 2009). The degree of lipid peroxidation is frequently assessed by measuring the formation of thiobarbituric acid reactive substances in plasma using the MDA of the total TBARS assays. In male elite football players, MDA and TBARS values are consistently reported to increase immediately after football matches and remain elevated up to 48–72 h postgame (Ascensao et al., 2008; Ispirlidis et al., 2008; Fatouros et al., 2010; Magalhães et al., 2010; Costa et al., 2011; Silva et al., 2013; Mohr et al., 2016; Koziol et al., 2020), which makes them an attractive biomarker to evaluate lipid oxidative damage during post-match recovery. However, these methods are subject to criticism as they present high cross-reactivity and might provide limited information on lipid peroxidation processes. Regarding serum proteins, these are the main responsible for scavenging free radicals, which in turn can lead to the formation of protein carbonyls as a sign of oxidative damage (Fisher-Wellman and Bloomer, 2009). Football matches have been reported to induce increases in protein carbonyls measurements from 0 to 72 h into the postgame with peak levels being observed at 48 h (Ispirlidis et al., 2008; Fatouros et al., 2010; Mohr et al., 2016; Souglis et al., 2018). Finally, the assessment of 8-OH-Dg as a sign of oxidative damage to DNA is scarcely reported in elite football research (Koziol et al., 2020). Concerning sex differences, measurements of oxidative stress and damage are typically lower in female compared with male athletes (Bloomer and Fisher-Wellman, 2008; Souglis et al., 2018).

Based on the above-mentioned studies, football matches might cause a pro-oxidative insult in semi-professional and professional football players denoted by increased levels of oxidated molecules and upregulation of antioxidative systems. If this pro-oxidative status can accumulate throughout subsequent matches is not obvious as studies evaluating the impact of congestive match schedules on different oxidative markers have yielded inconsistent results (Mohr et al., 2016; Poulios et al., 2018; Povoas et al., 2022). Finally, all available categories of oxidative biomarkers present important limitations that make difficult their individual application to sports practice, with the simultaneous measurement of multiple biomarkers being recommended (Powers and Jackson, 2008).

## 4 Role of sportomics in post-match recovery

As commented, serum creatine kinase is the most widely used biomarker of recovery in competitive sports such as football as it remains elevated several days into post-match recovery denoting the occurrence of EIMD (Mougios, 2007). However, post-match fatigue is a complex issue where mechanisms beyond EIMD involving central, endocrine, and metabolic disturbances partake, and the assessment of a single analyte might provide limited information. While several authors make a case for the use of biomarkers panels to overcome this problem, all commented biomarkers present limitations that make difficult their practical use. A summary of discussed biomarkers is presented in Table 1.

**TABLE 1:** Summary of available biomarkers of football post-match recovery.

Outcome	Biomarkers	Rationale for monitoring	Evidence	Main limitations
Muscle damage	CK, LDH, ALT, AST, Mb.	These analytes are released to the systemic circulation as a consequence of membrane disruption produced by EIMD, which is linked to reduced muscle power output and impaired gluconeogenesis.	While Mb levels tend to normalize within hours following football match-play, CK and LDH levels tend to remain elevated 48–72 h after matches and match simulation protocols.	Although CK is the most widely explored biomarker of EIMD, values are subject to high inter-individual variability.
			Increases in ALT and AST have been reported after matches, yet limited research on time-course analyses is available.	LDH, ALT, and AST are not as specific to the myocyte as CK, which makes them less reliable biomarkers for assessing muscle damage.
				Measuring Mb levels is useful for detecting acute muscle damage right after a football match; however, it is of scarce utility for monitoring long-term post-match recovery (i.e., 24–72 h).
Inflammation	CRP, IL-6, IL-1β, TNF-α.	The structural disarrangements that characterize EIMD are followed by an inflammatory response denoted by increased levels of these markers.	Large increases in IL-6 usually occur during the immediate postgame and normalize within hours.	Rather than EIMD, increases in IL-6 seem to reflect the skeletal muscle metabolic demands after a soccer match.
			CRP circulating levels tend to be impacted by soccer matches and peak at 24 h postgame, being base values restored within 72 h of recovery time.	Increases in CRP levels are not specific to exercise and may also occur due to clinical conditions.
			There is insufficient evidence on changes in cytokines such as IL-1β, or TNF-α during post-match recovery.	Changes in IL-1β and TNF-α have been insufficiently explored and results remain inconsistent.
Immune response	WBC counts, neutrophiles, monocytes, lymphocytes, sIgA.	WBC counts, particularly lymphocyte levels, as well as sIgA concentrations have been shown to decrease days after a football match which might increase the risk of infection and compromise recovery.	Match-induced perturbations in total WBC counts are usually restored within 24–48 h of recovery time.	Decrements in WBC counts might not represent immunosuppression but denote surveillance processes.
			Match congested fixture periods have been shown to decrease lymphocyte levels below resting values, but further research is needed.	Sampling procedure and the high interindividual heterogeneity might preclude the information provided by IgA measurements.
			Reported changes in salivary IgA values in response to football match-play have been shown unreliable for the monitoring of recovery.	
Endocrine response	Cortisol, testosterone.	Increased cortisol levels have been proposed to impair performance throughout post-match recovery due to enhanced catabolic processes while testosterone is thought to counterbalance cortisol actions.	Cortisol levels tend to increase during the immediate postgame with only some studies reporting sustained increases 48–72 h after.	There is currently insufficient evidence to recommend monitoring hormonal changes in high-level football players beyond the immediate post-match.
			Testosterone levels are frequently shown to decrease after the match, yet time-course analyses usually report unchanged levels during post-match recovery.	Research on hormonal changes in female football players in response to match-play is limited and might not mirror changes observed in male players.
Oxidative status	UA, GSH, GSSG, CAT, SOD, GPX, MDA, TBARS, PC, 8-OH-Dg.	Prolonged and damage-inducing exercise can lead to oxidative stress, which in turn has been linked to reduced muscle force production and aggravated EIMD, and is denoted by changes in circulating levels of oxidated and antioxidative molecules.	Plasma UA levels have been shown to increase right after a football match and can remain elevated days after.	Multiple methods are available, and the lack of reference values make comparisons and interpretation of results difficult.
			A decrease in the GSH/GSSG ratio within 24h–48 h after a football match might be indicative of oxidative stress associated to match-induced inflammatory processes.	Methodological challenges linked to available techniques might compromise the information provided by these compounds and their implementation in routine protocols.
			CAT activity is consistently reported to increase after the game and return to base values within hours. Postgame changes in SOD and GPX activity have been shown heterogeneous.	Other methods such as the assessment of TAC, TAS, ORAC, and FRAP are unspecific and neglect the role of intracellular antioxidant enzymes.
			The analysis of TBARS/MDA, protein carbonyls, and 8-OH-Dg can provide insights into lipid, protein, and nucleic acid oxidation processes respectively, 2–3 days into recovery from football match-play.	

8-OH-Dg, 8-hydroxy-2-deoxyguanosine; ALT, alanine transaminase; AST, aspartate transaminase; CAT, catalase; CK, creatine kinase; CRP, c-reactive protein; EIMD, exercise-induced muscle damage; FRAP, ferric reducing antioxidant potential; GPX, glutathione peroxidase; GSH, glutathione; GSSG, glutathione disulfide; IL, interleukin; LDH, lactate dehydrogenase; Mb, myoglobin; MDA, malondialdehyde; ORAC, oxygen radical antioxidant capacity; PC, protein carbonyls; sIgA, salivary immunoglobulin A; SOD, superoxide dismutase; TAC, total antioxidant capacity; TAS, total antioxidant status; TBARS, thiobarbituric acid reactive substances; TNF-α, tumor necrosis factor-α; UA, uric acid; WBC, white blood cells.

In addition to commented limitations, some practical aspects related to the monitoring of these biomarkers should be noted. The assessment of most available biomarkers relies on the analysis of venous or capillary blood samples through laboratory tests or point-of-care platforms (Sharma et al., 2021). Although the analysis of capillary blood permits a simple, inexpensive, and less invasive sampling process, significant heterogeneities between capillary and venous biomarker levels have been documented (Reichel et al., 2023). Additionally, some analytes, such cortisol and testosterone, are subject to important circadian variations, highlighting the importance of considering the timing of sampling (Gabriel and Zierath, 2019). Changes in inflammatory and immune biomarkers might reflect infection and other non-exercise-related inflammatory processes, so evaluating the player’s health status is important when monitoring these markers (Lee et al., 2017). Further, dehydration has been shown to exacerbate changes in recovery biomarkers (Ozkan and Ibrahim, 2016), which highlights the importance of monitoring hydration status through the assessment of bodily fluids and gross hydration markers [extensively reviewed elsewhere (Barley et al., 2020)]. Lastly, as previously commented, collection procedures of unstimulated saliva samples should consider important aspects such as oral cavity health status and saliva flow rate for the accurate measurement of salivary analytes (Djaoui et al., 2017). To address some of these issues, repeated testing using the same sample type in similar healthy states to establish personalized reference values, and further research in novel biomarkers that might provide more consistent information have been recommended (Lee et al., 2017).

In the last decades, there has been a dearth of research pertaining to the evaluation of novel biomarkers of recovery from exercise, and the analysis of available biomarkers has been suggested to only provide a narrow picture of the complex changes that the human body undergoes during exercise (San-Millan, 2019; Khoramipour et al., 2022). The application of metabolomic techniques to the field of sports, termed “sportomics,” holds promise for the non-invasive comprehensive characterization of the physiological changes that occur in response to exercise.

Sportomics is a holistic approach which encompasses the simultaneous analysis of numerous metabolites through the use of analytic techniques, such as gas and liquid chromatography, mass spectrometry, and nuclear magnetic resonance in biological samples in combination with data processing methods to investigate the metabolic changes induced by exercise (Bongiovanni et al., 2019). These analyses are classified into targeted metabolomics, if a selected number of metabolites of interest are evaluated, and non-targeted metabolomics, when a global approach is employed (Khoramipour et al., 2022). In fact, an increasing number of metabolomics studies has evaluated the metabolic profile of male and female high-level football players in different settings (Al-Muraikhy et al., 2021; Alzharani et al., 2020; Cicero et al., 2016; Marinho et al., 2022; Pintus et al., 2021; Pitti et al., 2019; Prado et al., 2017; Quintas et al., 2020; Santone et al., 2014; da Cruz et al., 2022; Luti et al., 2022). In particular, metabolomic analyses have been conducted in plasma, urine, and saliva samples to characterize the metabolic impact of training sessions (Cicero et al., 2016; Alzharani et al., 2020; Zhao et al., 2020), training programs (Pintus et al., 2021), football matches (Prado et al., 2017; Pitti et al., 2019), and entire competitive sessions in elite football players (Quintas et al., 2020).

One of the first studies focused on football match-induced fatigue was conducted by Ra et al. (2014) who compared the non-targeted salivary metabolic profile of 37 fatigued intercollegiate male football players selected based on changes in heart rate, body mass index, and psychological tests scores before and after three games played in three consecutive days. Authors concluded that the consecutive matches program induced variations in protein breakdown markers such as 3-methylhistidine, and amino acids as well as metabolites related to glycolytic and gluconeogenic pathways (Ra et al., 2014). An ensuing study conducted by Pitti et al. (2019) observed that a single official football match was capable of eliciting changes in amino acids and energy metabolites salivary profiles in female professional players (Pitti et al., 2019). However, aligned with reviews on metabolomics studies (Schranner et al., 2020), most of the studies conducted to this date have focused on the immediate or acute effects of exercise on the metabolic status of athletes while there is a scarcity of studies assessing different time-points during the post-match recovery period (i.e., at 24, 48, or 72 h).

On this subject, Zhao et al. (2020) conducted a non-targeted metabolomic and proteomic analysis of urine samples obtained before, 30 min and 18 h after performing a high intensity interval training in 23 professional players from a youth team. While changes in amino acid metabolism pathways, purine cycle metabolism, and different analytes related to the energy metabolism were present from pre-to post-exercise time-points, most of these changes returned to pre-exercise values during recovery. On the contrary, steroids hormones were downregulated during post-exercise and upregulated at 18 h. Furthermore, the proteomic analysis revealed that proteins linked to energy production and defense mechanisms, among others, were upregulated after exercise and downregulated during recovery (Zhao et al., 2020). Recently, Marinho et al. (2022) studied the metabolic impact that two friendly football matches separated by 72 h had in elite male U20 players through the assessment of urine samples collected immediately and 20 h after both matches. According to the authors, while changes in lipids, proteins and energy pathways were present immediately after the matches, alterations in levels of metabolites related to anti-inflammatory and anti-oxidant processes predominated at 20 h postgame, which were more pronounced after the second match (Marinho et al., 2022). To this date, only one study conducted in elite rugby has explored the blood, urine, and saliva un-targeted metabolomic profile of players during the 24–48 h post-match recovery period. Between 24 and 48 h after the match, a shift towards increased gluconeogenesis and glycolysis, higher protein degradation, impaired fatty acid metabolism, and oxidative processes was observed, which was proposed to denote metabolic disturbances caused by EIMD (Hudson et al., 2021). Similar approaches applied to football match-play would improve our knowledge on the metabolic needs of players during post-match recovery.

## 5 Future perspectives

Long time-course analyses in sportomics studies are needed but face various challenges. Future studies should carefully monitor dietary intake and training sessions during post-match recovery to ensure the ecological validity of observed data (Close et al., 2019). Adherence to nutritional guidelines for both match-day and post-match recovery, and the standardization of training loads are key for achieving this goal (Mohr et al., 2016). These studies would also benefit from evaluating non-invasive samples such as saliva and urine to improve the applicability to real-world conditions (Bongiovanni et al., 2022). Additionally, systematic analyses have shown that sex plays an important role in driving differences in metabolomic profiles, thus representing an important factor to consider in football studies (Costanzo et al., 2022).

In the same line, sex-based differences in match loads and activity patterns have been reported, yet few studies have explored time-course differences in circulating biomarkers during post-match recovery in elite female players, in alignment with a recent meta-analysis (Goulart et al., 2022). On this subject, the impact of menstrual cycle phases on recovery patterns deserves further research (Goulart et al., 2022). Another potential avenue for future research is to investigate whether the algorithms that are currently used to personalize CK values in football players can also be applied to other discussed biomarkers as well as to physical and perceptual measurements (Skorski et al., 2022).

## 6 Conclusion

Football match-play puts a physical strain on high-level football players denoted by a decline in physical performance that can persist over days. The monitoring of biomarkers provides an objective measurement of the players’ internal load during post-match recovery, which might supplement the assessment of functional and subjective indicators. However, no single biomarker can delineate the complex processes that comprise exercise recovery and aspects such as timing, sample type, technical limitations, inter-individual variance, and contextual factors make challenging the extrapolation of data reported in single studies. The use of biomarkers panels, and the individualization of physiological ranges might provide a more robust assessment of players’ fatigue during recovery, yet further research on fluctuations of different analytes throughout post-match recovery is warranted. The application of metabolomics to football research might support the identification of novel biomarkers and might also provide information on individual’s metabolomic profile to guide personalized recovery protocols. Nonetheless, most metabolomics studies conducted to date have focused on acute fatigue processes thus neglecting the long-term impact of football competition. Future studies might benefit from differentiating the metabolic disturbances that characterize the acute and residual post-match fatigue response.
