# Supplementary material for: Biomarkers of post-match recovery in semi-professional and professional football (soccer)
Source: Front Physiol. 2023 Apr 11;14:1167449. doi: 10.3389/fphys.2023.1167449 (PMC10126523; doi:10.3389/fphys.2023.1167449)
Supplement: Supplementary file 1 [file Table1.DOCX]

**Supplementary 1**

**TABLE S1** Time-course analyses of biomarkers of exercise-induced muscle damage in professional and semi-professional football

| Marker | Sex | Reference | Protocol | MD | MD+1 | MD+2 | MD+3 |
| --- | --- | --- | --- | --- | --- | --- | --- |
| CK | Male | (Ascensao et al., 2008) | Match |  |  |  |  |
|  |  | (Ispirlidis et al., 2008) | Match |  |  |  |  |
|  |  | (Magalhães et al., 2010) | LIST |  |  |  |  |
|  |  | (Magalhães et al., 2010) | Match |  |  |  |  |
|  |  | (Fatouros et al., 2010) | Match |  |  |  |  |
|  |  | (Rampinini et al., 2011) | Match |  |  |  |  |
|  |  | (Gunnarsson et al., 2013) | Match | **=** |  | **=** |  |
|  |  | (Silva et al., 2013) | Match |  |  |  | **=** |
|  |  | (Romagnoli et al., 2016) | Match |  |  |  |  |
|  |  | (Souglis et al., 2015a)** | Match |  |  |  |  |
|  |  | (Souglis et al., 2015b) | Match |  |  |  |  |
|  |  | (Mohr et al., 2016) (Match 1) | Match |  |  |  |  |
|  |  | (Mohr et al., 2016) (Match 2) | Match |  |  |  |  |
|  |  | (Mohr et al., 2016) (Match 3) | Match |  |  |  |  |
|  |  | (Fransson et al., 2018) | CST | **=** |  | **=** |  |
|  |  | (Bouzid et al., 2019) | LIST | **=** |  | **=** | **=** |
|  |  | (Koziol et al., 2020) | Match |  |  |  |  |
|  |  | (Daab et al., 2021) | LIST |  |  |  | **=** |
|  |  | (AdibSaber et al., 2022) | Match |  |  |  |  |
|  |  | (Wiig et al., 2022) | Match |  |  |  |  |
|  | Female | (Andersson et al., 2008)*** | Match |  |  |  | **=** |
|  |  | (Gravina et al., 2011)* | Match | **=** |  |  |  |
|  |  | (Souglis et al., 2015b) | Match |  |  |  |  |
|  |  | (Gomes et al., 2018) | Match |  |  |  |  |
| LDH | Male | (Ispirlidis et al., 2008) | Match |  |  |  |  |
|  |  | (Souglis et al., 2015a)** | Match |  |  |  |  |
|  |  | (Daab et al., 2021) | LIST |  |  |  |  |
|  |  | (AdibSaber et al., 2022) | Match |  |  |  |  |
|  | Female | (Gravina et al., 2011)* | Match |  | **=** |  |  |
| ALT & AST | Male | (AdibSaber et al., 2022) | Match |  |  |  |  |
|  | Female | (Gravina et al., 2011)* | Match | **=** | **=** |  |  |
|  |  | (Gomes et al., 2018) | Match | **=** |  | **=** |  |
| Myoglobin | Male | (Ascensao et al., 2008) | Match |  | **=** | **=** | **=** |
|  |  | (Magalhães et al., 2010) | LIST |  | **=** | **=** | **=** |
|  |  | (Magalhães et al., 2010) | Match |  | **=** | **=** | **=** |
|  |  | (Gunnarsson et al., 2013) | Match |  | **=** | **=** |  |
|  |  | (Silva et al., 2013) | Match |  |  | **=** | **=** |
|  |  | (Fransson et al., 2018) | CST |  | **=** | **=** |  |
|  |  | (Wiig et al., 2022) | Match |  |  | **=** | **=** |

* Measured at 18h, ** measured at 0h, 13h, and 37h, *** measured at 21h, 45h, and 69h, statistically significant increase compared with baseline or control, statistically significant decrease compared with baseline or control, **=** no statistically significant difference compared with baseline or control, ALT alanine transaminase, AST aspartate transaminase, CK creatine kinase, CST Copenhagen Soccer Test, LDH lactate dehydrogenase, LIST Loughborough Intermittent Shuttle Test, MD match day.

**TABLE S2** Time-course analyses of biomarkers of inflammation in professional and semi-professional football

| Marker | Sex | Reference | Protocol | MD | MD+1 | MD+2 | MD+3 |
| --- | --- | --- | --- | --- | --- | --- | --- |
| IL-6 | Male | (Ispirlidis et al., 2008) | Match |  | **=** | **=** | **=** |
|  |  | (Romagnoli et al., 2016) | Match |  | **=** | **=** |  |
|  |  | (Souglis et al., 2015a)** | Match |  |  | **=** |  |
|  |  | (Souglis et al., 2015b) | Match |  | **=** | **=** |  |
|  |  | (Mohr et al., 2016) (Match 1) | Match | **=** | **=** | **=** | **=** |
|  |  | (Mohr et al., 2016) (Match 2) | Match | **=** | **=** | **=** | **=** |
|  |  | (Mohr et al., 2016) (Match 3) | Match | **=** | **=** | **=** | **=** |
|  |  | (Koziol et al., 2020) | Match |  | **=** |  |  |
|  | Female | (Andersson et al., 2010a) | Match |  | **=** | **=** | **=** |
|  |  | (Souglis et al., 2015b) | Match |  | **=** | **=** |  |
| IL-1β | Male | (Mohr et al., 2016) (Match 1) | Match | **=** | **=** | **=** | **=** |
|  |  | (Mohr et al., 2016) (Match 2) | Match | **=** | **=** | **=** | **=** |
|  |  | (Mohr et al., 2016) (Match 3) | Match | **=** | **=** | **=** | **=** |
|  | Female | (Andersson et al., 2010a) | Match | **=** | **=** | **=** | **=** |
| TNF-α | Male | (Souglis et al., 2015a)** | Match |  |  | **=** |  |
|  |  | (Souglis et al., 2015b) | Match |  | **=** | **=** |  |
|  |  | (Koziol et al., 2020) | Match |  | **=** |  |  |
|  | Female | (Andersson et al., 2010a) | Match |  | **=** | **=** | **=** |
| CRP | Male | (Ispirlidis et al., 2008) | Match |  |  | **=** | **=** |
|  |  | (Silva et al., 2013) | Match |  |  | **=** | **=** |
|  |  | (Souglis et al., 2015a)** | Match |  |  | **=** |  |
|  |  | (Souglis et al., 2015b) | Match | **=** |  | **=** | **=** |
|  |  | (Mohr et al., 2016) (Match 1) | Match |  |  | **=** | **=** |
|  |  | (Mohr et al., 2016) (Match 2) | Match |  |  |  | **=** |
|  |  | (Mohr et al., 2016) (Match 3) | Match |  |  | **=** | **=** |
|  |  | (Romagnoli et al., 2016) | Match | **=** |  | **=** |  |
|  |  | (Fransson et al., 2018) | CST | **=** | **=** | **=** |  |
|  |  | (Daab et al., 2021) | LIST | **=** |  |  | **=** |
|  |  | (Duarte et al., 2022) (Match 1) | Match | **=** |  |  | **=** |
|  |  | (Duarte et al., 2022) (Match 2) | Match | **=** |  |  | **=** |
|  |  | (Duarte et al., 2022) (Match 3) | Match | **=** |  |  | **=** |
|  | Female | (Gravina et al., 2011)* | Match | **=** |  |  |  |
|  |  | (Souglis et al., 2015b) | Match | **=** |  | **=** | **=** |
|  |  | (Goulart et al., 2021) | Match |  | **=** | **=** | **=** |

* Measured at 18h, ** measured at 0h, 13h, and 37h, statistically significant increase compared with baseline or control, statistically significant decrease compared with baseline or control, **=** no statistically significant difference compared with baseline or control, CRP C-reactive protein, CST Copenhagen Soccer Test, IL interleukin, LDH lactate dehydrogenase, LIST Loughborough Intermittent Shuttle Test, MD match day, TNF-α tumor necrosis factor-α.

**TABLE S3** Time-course analyses of biomarkers of immune response in professional and semi-professional football

| Marker | Sex | Reference | Protocol | MD | MD+1 | MD+2 | MD+3 |
| --- | --- | --- | --- | --- | --- | --- | --- |
| WBC counts | Male | (Ascensao et al., 2008) | Match |  | **=** | **=** | **=** |
|  |  | (Ispirlidis et al., 2008) | Match |  |  | **=** | **=** |
|  |  | (Fatouros et al., 2010) | Match |  |  | **=** | **=** |
|  |  | (Magalhães et al., 2010) | LIST |  | **=** | **=** | **=** |
|  |  | (Magalhães et al., 2010) | Match |  | **=** | **=** | **=** |
|  |  | (Mohr et al., 2016) (Match 1) | Match |  |  | **=** | **=** |
|  |  | (Mohr et al., 2016) (Match 2) | Match |  |  | **=** | **=** |
|  |  | (Mohr et al., 2016) (Match 3) | Match |  |  | **=** | **=** |
|  |  | (Romagnoli et al., 2016) | Match |  | **=** | **=** |  |
|  | Female | (Gravina et al., 2011)* | Match |  | **=** |  |  |
| Neutrophiles | Male | (Ascensao et al., 2008) | Match |  | **=** | **=** | **=** |
|  |  | (Romagnoli et al., 2016) | Match |  |  |  |  |
|  | Female | (Gravina et al., 2011) | Match |  | **=** |  |  |
| Monocytes | Male | (Romagnoli et al., 2016) | Match |  | **=** | **=** |  |
|  | Female | (Gravina et al., 2011)* | Match | **=** | **=** |  |  |
| Lymphocytes | Male | (Ascensao et al., 2008) | Match |  | **=** | **=** | **=** |
|  |  | (Magalhães et al., 2010) | LIST |  | **=** | **=** | **=** |
|  |  | (Magalhães et al., 2010) | Match |  | **=** | **=** | **=** |
|  |  | (Romagnoli et al., 2016) | Match | **=** | **=** | **=** |  |
|  | Female | (Gravina et al., 2011)* | Match |  | **=** |  |  |

* Measured at 18h, statistically significant increase compared with baseline or control, statistically significant decrease compared with baseline or control, **=** no statistically significant difference compared with baseline or control, LIST Loughborough Intermittent Shuttle Test, MD match day, WBC white blood cells.

**TABLE S4** Time-course analyses of biomarkers of endocrine response in professional and semi-professional football

| Marker | Sex | Reference | Protocol | MD | MD+1 | MD+2 | MD+3 |
| --- | --- | --- | --- | --- | --- | --- | --- |
| Cortisol | Male | (Ispirlidis et al., 2008) | Match |  | **=** | **=** | **=** |
|  |  | (Silva et al., 2013) | Match |  |  |  | **=** |
|  |  | (Souglis et al., 2015a)** | Match |  | **=** | **=** |  |
|  |  | (Romagnoli et al., 2016) | Match | **=** |  |  |  |
|  |  | (Mohr et al., 2016) (Match 1) | Match |  | **=** | **=** | **=** |
|  |  | (Mohr et al., 2016) (Match 2) | Match |  |  | **=** | **=** |
|  |  | (Mohr et al., 2016) (Match 3) | Match |  | **=** | **=** | **=** |
|  |  | (Koziol et al., 2020) | Match |  | **=** |  |  |
|  |  | (Morgans et al., 2022) (Saliva) | Match |  |  |  |  |
| Testosterone | Male | (Ispirlidis et al., 2008) | Match | **=** | **=** | **=** | **=** |
|  |  | (Silva et al., 2013) | Match |  | **=** | **=** | **=** |
|  |  | (Mohr et al., 2016) (Match 1) | Match | **=** | **=** | **=** | **=** |
|  |  | (Mohr et al., 2016) (Match 2) | Match | **=** | **=** | **=** | **=** |
|  |  | (Mohr et al., 2016) (Match 3) | Match | **=** | **=** | **=** | **=** |
|  |  | (Romagnoli et al., 2016) | Match |  |  |  |  |
|  | Female | (Gravina et al., 2011)* | Match |  | **=** |  |  |

* Measured at 18h, ** measured at 0h, 13h, and 37h, statistically significant increase compared with baseline or control, statistically significant decrease compared with baseline or control, **=** no statistically significant difference compared with baseline or control, LIST Loughborough Intermittent Shuttle Test, MD match day.

**TABLE S5** Time-course analyses of biomarkers of oxidative status in professional and semi-professional football

| Marker | Sex | Reference | Protocol | MD | MD+1 | MD+2 | MD+3 |
| --- | --- | --- | --- | --- | --- | --- | --- |
| UA | Male | (Ascensao et al., 2008) | Match |  |  |  |  |
|  |  | (Ispirlidis et al., 2008) | Match | **=** |  |  |  |
|  |  | (Fatouros et al., 2010) | Match | **=** |  |  | **=** |
|  |  | (Magalhães et al., 2010) | LIST |  | **=** | **=** | **=** |
|  |  | (Magalhães et al., 2010) | Match |  | **=** | **=** | **=** |
|  |  | (Silva et al., 2013) | Match |  | **=** | **=** | **=** |
|  |  | (Bouzid et al., 2019) | LIST |  |  | **=** | **=** |
|  | Female | (Andersson et al., 2008) | Match |  | **=** | **=** | **=** |
|  |  | (Andersson et al., 2010b) | Match |  | **=** | **=** | **=** |
|  |  | (Andersson et al., 2010c)**** | Match |  | **=** |  |  |
|  |  | (Gravina et al., 2011)* | Match |  | **=** |  |  |
|  |  | (Gomes et al., 2018) | Match | **=** |  |  |  |
|  |  | (Gomes et al., 2018) (Saliva) | Match | **=** |  | **=** |  |
| GSH | Male | (Fatouros et al., 2010) | Match | **=** |  | **=** | **=** |
|  |  | (Mohr et al., 2016) (Match 1) | Match |  |  |  | **=** |
|  |  | (Mohr et al., 2016) (Match 2) | Match |  |  |  | **=** |
|  |  | (Mohr et al., 2016) (Match 3) | Match |  |  |  | **=** |
|  |  | (Koziol et al., 2020) | Match |  |  |  |  |
|  | Female | (Andersson et al., 2010b) | Match | **=** | **=** |  |  |
|  |  | (Gomes et al., 2018) | Match | **=** |  | **=** |  |
| GSSG | Male | (Fatouros et al., 2010) | Match | **=** |  |  | **=** |
|  |  | (Mohr et al., 2016) (Match 1) | Match | **=** |  | **=** | **=** |
|  |  | (Mohr et al., 2016) (Match 2) | Match | **=** |  |  | **=** |
|  |  | (Mohr et al., 2016) (Match 3) | Match | **=** |  |  | **=** |
|  |  | (Koziol et al., 2020) | Match |  |  |  |  |
|  | Female | (Gomes et al., 2018) | Match |  |  |  |  |
| CAT | Male | (Fatouros et al., 2010) | Match |  | **=** | **=** | **=** |
|  |  | (Mohr et al., 2016) (Match 1) | Match |  | **=** | **=** | **=** |
|  |  | (Mohr et al., 2016) (Match 2) | Match |  | **=** | **=** | **=** |
|  |  | (Mohr et al., 2016) (Match 3) | Match |  | **=** | **=** | **=** |
| SOD | Male | (Silva et al., 2013) | Match |  |  |  | **=** |
|  | Female | (Gravina et al., 2011)* | Match | **=** | **=** |  |  |
| GPX | Male | (Fatouros et al., 2010) | Match | **=** |  |  |  |
|  |  | (Silva et al., 2013) | Match |  |  | **=** | **=** |
|  |  | (Mohr et al., 2016) (Match 1) | Match |  |  |  |  |
|  |  | (Mohr et al., 2016) (Match 2) | Match |  |  |  |  |
|  |  | (Mohr et al., 2016) (Match 3) | Match |  |  |  | **=** |
|  | Female | (Gravina et al., 2011) | Match | **=** | **=** |  |  |
| MDA/TBARS | Male | (Ascensao et al., 2008) | Match |  |  |  |  |
|  |  | (Ispirlidis et al., 2008) | Match |  |  |  | **=** |
|  |  | (Fatouros et al., 2010) | Match |  |  |  | **=** |
|  |  | (Magalhães et al., 2010) | LIST |  |  |  |  |
|  |  | (Magalhães et al., 2010) | Match |  |  |  |  |
|  |  | (Silva et al., 2013) | Match |  |  |  | **=** |
|  |  | (Mohr et al., 2016) (Match 1) | Match |  |  |  |  |
|  |  | (Mohr et al., 2016) (Match 2) | Match |  |  |  |  |
|  |  | (Mohr et al., 2016) (Match 3) | Match |  |  |  |  |
|  |  | (Koziol et al., 2020) | Match | **=** |  |  |  |
| PC | Male | (Ispirlidis et al., 2008) | Match |  |  |  |  |
|  |  | (Fatouros et al., 2010) | Match |  |  |  |  |
|  |  | (Mohr et al., 2016) (Match 1) | Match |  |  |  |  |
|  |  | (Mohr et al., 2016) (Match 2) | Match |  |  |  |  |
|  |  | (Mohr et al., 2016) (Match 3) | Match |  |  |  | **=** |
| 8-OH-Dg | Male | (Koziol et al., 2020) | Match | **=** |  |  |  |

* Measured at 18h, ** measured at 0h, 13h, and 37h, **** measured at 21h, statistically significant increase compared with baseline or control, statistically significant decrease compared with baseline or control, **=** no statistically significant difference compared with baseline or control, 8-OH-Dg 8-hydroxy-2-deoxyguanosine, CAT catalase, GPX glutathione peroxidase, GSH glutathione, GSSG glutathione disulfide, MDA malondialdehyde, LIST Loughborough Intermittent Shuttle Test, PC protein carbonyls, SOD superoxide dismutase, TBARS thiobarbituric acid reactive substances, UA uric acid.

**References**

ADIBSABER, F., ANSARI, S., ELMIEH, A. & RAJABZADEH, H. 2022. Effect of an Energy Drink On Muscle and Liver Damage Enzymes, And Cardiovascular Indices in Soccer Players. *Sci Med Footb***,** 1-7.

ANDERSSON, H., BØHN, S. K., RAASTAD, T., PAULSEN, G., BLOMHOFF, R. & KADI, F. 2010a. Differences in the inflammatory plasma cytokine response following two elite female soccer games separated by a 72-h recovery. *Scandinavian Journal of Medicine & Science in Sports,* 20**,** 740-747.

ANDERSSON, H., KARLSEN, A., BLOMHOFF, R., RAASTAD, T. & KADI, F. 2010b. Active recovery training does not affect the antioxidant response to soccer games in elite female players. *Br J Nutr,* 104**,** 1492-9.

ANDERSSON, H., KARLSEN, A., BLOMHOFF, R., RAASTAD, T. & KADI, F. 2010c. Plasma antioxidant responses and oxidative stress following a soccer game in elite female players. *Scand J Med Sci Sports,* 20**,** 600-8.

ANDERSSON, H., RAASTAD, T., NILSSON, J., PAULSEN, G., GARTHE, I. & KADI, F. 2008. Neuromuscular fatigue and recovery in elite female soccer: effects of active recovery. *Med Sci Sports Exerc,* 40**,** 372-80.

ASCENSAO, A., REBELO, A., OLIVEIRA, E., MARQUES, F., PEREIRA, L. & MAGALHAES, J. 2008. Biochemical impact of a soccer match - analysis of oxidative stress and muscle damage markers throughout recovery. *Clin Biochem,* 41**,** 841-51.

BOUZID, M. A., ABAIDIA, A. E., BOUCHIBA, M., GHATTASSI, K., DAAB, W., ENGEL, F. A. & CHTOUROU, H. 2019. Effects of Ramadan Fasting on Recovery Following a Simulated Soccer Match in Professional Soccer Players: A Pilot Study. *Front Physiol,* 10**,** 1480.

DAAB, W., BOUZID, M. A., LAJRI, M., BOUCHIBA, M., SAAFI, M. A. & REBAI, H. 2021. Chronic Beetroot Juice Supplementation Accelerates Recovery Kinetics following Simulated Match Play in Soccer Players. *J Am Coll Nutr,* 40**,** 61-69.

DUARTE, W., RODRIGUES JÚNIOR, J. L., PAULA, L. V., CHAGAS, M. H., ANDRADE, A. G. P., VENEROSO, C. E., CHAVES, S. F. N., SERPA, T. K. F. & PIMENTA, E. M. 2022. C-Reactive Protein and Skin Temperature of the lower limbs of Brazilian elite soccer players like load markers following three consecutive games. *Journal of Thermal Biology,* 105**,** 103188.

FATOUROS, I. G., CHATZINIKOLAOU, A., DOUROUDOS, I. I., NIKOLAIDIS, M. G., KYPAROS, A., MARGONIS, K., MICHAILIDIS, Y., VANTARAKIS, A., TAXILDARIS, K., KATRABASAS, I., MANDALIDIS, D., KOURETAS, D. & JAMURTAS, A. Z. 2010. Time-Course of Changes in Oxidative Stress and Antioxidant Status Responses Following a Soccer Game. *The Journal of Strength & Conditioning Research,* 24.

FRANSSON, D., VIGH-LARSEN, J. F., FATOUROS, I. G., KRUSTRUP, P. & MOHR, M. 2018. Fatigue Responses in Various Muscle Groups in Well-Trained Competitive Male Players after a Simulated Soccer Game. *Journal of Human Kinetics,* 61**,** 85-97.

GOMES, D., ROSA-LIMA, F., MELLO, R., PAZ, G., MIRANDA, H. & SALERNO, V. 2018. Oxidative stress, muscle and liver cell damage in professional soccer players during a 2-game week schedule. *Science & Sports,* 33.

GOULART, K. N. O., COUTO, B. P., JUNIOR, G. O. C., PIMENTA, E. M. & DUFFIELD, R. 2021. The effect of post-match resistance training on recovery in female footballers; when is best to train? *Sci Med Footb,* 5**,** 208-215.

GRAVINA, L., RUIZ, F., LEKUE, J. A., IRAZUSTA, J. & GIL, S. M. 2011. Metabolic impact of a soccer match on female players. *J Sports Sci,* 29**,** 1345-52.

GUNNARSSON, T. P., BENDIKSEN, M., BISCHOFF, R., CHRISTENSEN, P. M., LESIVIG, B., MADSEN, K., STEPHENS, F., GREENHAFF, P., KRUSTRUP, P. & BANGSBO, J. 2013. Effect of whey protein- and carbohydrate-enriched diet on glycogen resynthesis during the first 48 h after a soccer game. *Scand J Med Sci Sports,* 23**,** 508-15.

ISPIRLIDIS, I., FATOUROS, I. G., JAMURTAS, A. Z., NIKOLAIDIS, M. G., MICHAILIDIS, I., DOUROUDOS, I., MARGONIS, K., CHATZINIKOLAOU, A., KALISTRATOS, E., KATRABASAS, I., ALEXIOU, V. & TAXILDARIS, K. 2008. Time-course of Changes in Inflammatory and Performance Responses Following a Soccer Game. *Clinical Journal of Sport Medicine,* 18.

KOZIOL, K., ZEBROWSKI, J., BETLEJ, G., BATOR, E., CZARNY, W., BAJOREK, W., CZARNOTA, B., CZAJA, R., KROL, P. & KWIATKOWSKA, A. 2020. Changes in gammaH2AX and H4K16ac levels are involved in the biochemical response to a competitive soccer match in adolescent players. *Sci Rep,* 10**,** 14481.

MAGALHÃES, J., REBELO, A., OLIVEIRA, E., SILVA, J. R., MARQUES, F. & ASCENSÃO, A. 2010. Impact of Loughborough Intermittent Shuttle Test versus soccer match on physiological, biochemical and neuromuscular parameters. *Eur J Appl Physiol,* 108**,** 39-48.

MOHR, M., DRAGANIDIS, D., CHATZINIKOLAOU, A., BARBERO-ALVAREZ, J. C., CASTAGNA, C., DOUROUDOS, I., AVLONITI, A., MARGELI, A., PAPASSOTIRIOU, I., FLOURIS, A. D., JAMURTAS, A. Z., KRUSTRUP, P. & FATOUROS, I. G. 2016. Muscle damage, inflammatory, immune and performance responses to three football games in 1 week in competitive male players. *Eur J Appl Physiol,* 116**,** 179-93.

MORGANS, R., ORME, P., BEZUGLOV, E., DI MICHELE, R. & MOREIRA, A. 2022. The Immunological and Hormonal Responses to Competitive Match-Play in Elite Soccer Players. *Int J Environ Res Public Health,* 19.

RAMPININI, E., BOSIO, A., FERRARESI, I., PETRUOLO, A., MORELLI, A. & SASSI, A. 2011. Match-related fatigue in soccer players. *Med Sci Sports Exerc,* 43**,** 2161-70.

ROMAGNOLI, M., SANCHIS-GOMAR, F., ALIS, R., RISSO-BALLESTER, J., BOSIO, A., GRAZIANI, R. L. & RAMPININI, E. 2016. Changes in muscle damage, inflammation, and fatigue-related parameters in young elite soccer players after a match. *J Sports Med Phys Fitness,* 56**,** 1198-1205.

SILVA, J. R., ASCENSAO, A., MARQUES, F., SEABRA, A., REBELO, A. & MAGALHAES, J. 2013. Neuromuscular function, hormonal and redox status and muscle damage of professional soccer players after a high-level competitive match. *Eur J Appl Physiol,* 113**,** 2193-201.

SOUGLIS, A., BOGDANIS, G. C., GIANNOPOULOU, I., PAPADOPOULOS, C. & APOSTOLIDIS, N. 2015a. Comparison of inflammatory responses and muscle damage indices following a soccer, basketball, volleyball and handball game at an elite competitive level. *Res Sports Med,* 23**,** 59-72.

SOUGLIS, A. G., PAPAPANAGIOTOU, A., BOGDANIS, G. C., TRAVLOS, A. K., APOSTOLIDIS, N. G. & GELADAS, N. D. 2015b. Comparison of inflammatory responses to a soccer match between elite male and female players. *J Strength Cond Res,* 29**,** 1227-33.

WIIG, H., CUMMING, K. T., HANDEGAARD, V., STABELL, J., SPENCER, M. & RAASTAD, T. 2022. Muscular heat shock protein response and muscle damage after semi-professional football match. *Scand J Med Sci Sports,* 32**,** 984-996.
